# NAD^+^-consuming enzymes in immune defense against viral infection

**DOI:** 10.1042/BCJ20210181

**Published:** 2021-12-06

**Authors:** Jialin Shang, Michael R. Smith, Ananya Anmangandla, Hening Lin

**Affiliations:** 1 Department of Chemistry and Chemical Biology, Cornell University, Ithaca, NY 14853, U.S.A.; 2 Howard Hughes Medical Institute, Department of Chemistry and Chemical Biology, Cornell University, Ithaca, NY 14853, U.S.A.

**Keywords:** immune response, macrodomain, NAD^+^-consuming enzymes, PARP, sirtuins, virus

## Abstract

The COVID-19 pandemic reminds us that in spite of the scientific progress in the past century, there is a lack of general antiviral strategies. In analogy to broad-spectrum antibiotics as antibacterial agents, developing broad spectrum antiviral agents would buy us time for the development of vaccines and treatments for future viral infections. In addition to targeting viral factors, a possible strategy is to understand host immune defense mechanisms and develop methods to boost the antiviral immune response. Here we summarize the role of NAD^+^-consuming enzymes in the immune defense against viral infections, with the hope that a better understanding of this process could help to develop better antiviral therapeutics targeting these enzymes. These NAD^+^-consuming enzymes include PARPs, sirtuins, CD38, and SARM1. Among these, the antiviral function of PARPs is particularly important and will be a focus of this review. Interestingly, NAD^+^ biosynthetic enzymes are also implicated in immune responses. In addition, many viruses, including SARS-CoV-2 contain a macrodomain-containing protein (NSP3 in SARS-CoV-2), which serves to counteract the antiviral function of host PARPs. Therefore, NAD^+^ and NAD^+^-consuming enzymes play crucial roles in immune responses against viral infections and detailed mechanistic understandings in the future will likely facilitate the development of general antiviral strategies.

NAD^+^ or nicotinamide adenine dinucleotide (Figure 1, center) is a co-factor or co-enzyme used in many metabolic reactions. Several enzymes in glycolysis and the Krebs cycle use NAD^+^ to generate NADH. NADH is mainly used in oxidative phosphorylation to generate ATP, during which NADH is oxidized back to NAD^+^. In cancer cells subject to the Warburg effect, NADH is also used to reduce pyruvate to generate NAD^+^ and lactate, making sure that the redox state is optimal for cancer cell proliferation [1]. Thus, NAD^+^ is a vital co-enzyme for cellular metabolism and it is important for cells to maintain proper NAD^+^ levels.

**Figure 1.**
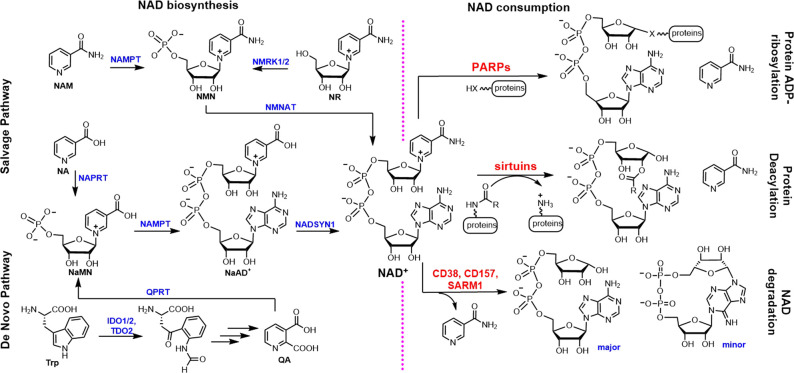
Scheme showing the structure of NAD^+^, NAD^+^-consuming reactions catalyzed by PARPs, sirtuins, and NAD^+^ glycohydrolases, and the NAD^+^ biosynthesis pathways. Abbreviation for small molecules: NAM, nicotinamide; NA, nicotinic acid; NaMN, nicotinate mononucleotide; NMN, nicotinamide mononucleotide; NaAD^+^, nicotinate adenine dinucleotide; NR, nicotinamide riboside; Trp, tryptophan; QA, quinolinic acid.

Given the importance of NAD^+^ as a co-enzyme, it is interesting and perhaps surprising that almost all eukaryotes, including mammals, have enzymes that consume NAD^+^ (Figure 1, right). These enzymes can be broadly classified into two categories: enzymes that break down NAD^+^ and transfer the ADP-ribosyl group to other proteins, and enzymes that hydrolyze NAD^+^ to ADP-ribose and nicotinamide.

The first category includes sirtuins, poly-ADP-ribose polymerases (PARPs), and extracellular mono-ADP-ribosyltransferases (ARTs). ARTs are ecto-enzymes catalyzing protein mono-ADP-ribosylation [2]. Their function is relatively little understood and thus will not be further discussed here.

Sirtuins were initially found to be important in gene silencing and calorie restriction-induced life span extension in model organisms [3]. Later studies showed that they couple NAD^+^ degradation to the deacylation (such as deacetylation, desuccinylation, and demyristoylation) [4–7] of various substrates proteins, which mediates the effects of sirtuins in gene silencing and other biological processes, such as regulation of metabolic enzymes and signaling proteins.

PARPs catalyze the addition of ADP-ribosyl groups from NAD^+^ to nucleophilic side-chains of protein, including aspartate, glutamate, serine, cysteine, and lysine [8,9]. Recent studies suggest that they could also ADP-ribosylate DNA and RNA [10]. The PARP family of enzymes, with 17 members in humans, shares a conserved catalytic domain, divided into five subfamilies according to domain structure and function (Figure 2) [11]. Though initially believed to catalyze primarily the transfer of linear or branched poly-ADP-ribose (PAR) or PARylation, recent research showed that many PARPs catalyze mono-ADP-ribosylation (MARylation). PARPs vary in their transcriptional level and cellular localization, and contain a broad range of regulatory domains which allow them to participate in diverse cellular functions [12]. The best-characterized functions involve regulation of stress responses such as DNA damage, apoptosis, and unfolded protein response [13–16]. However, emerging roles in pathogen response and non-stress related regulatory roles have been described [17–22].

**Figure 2.**
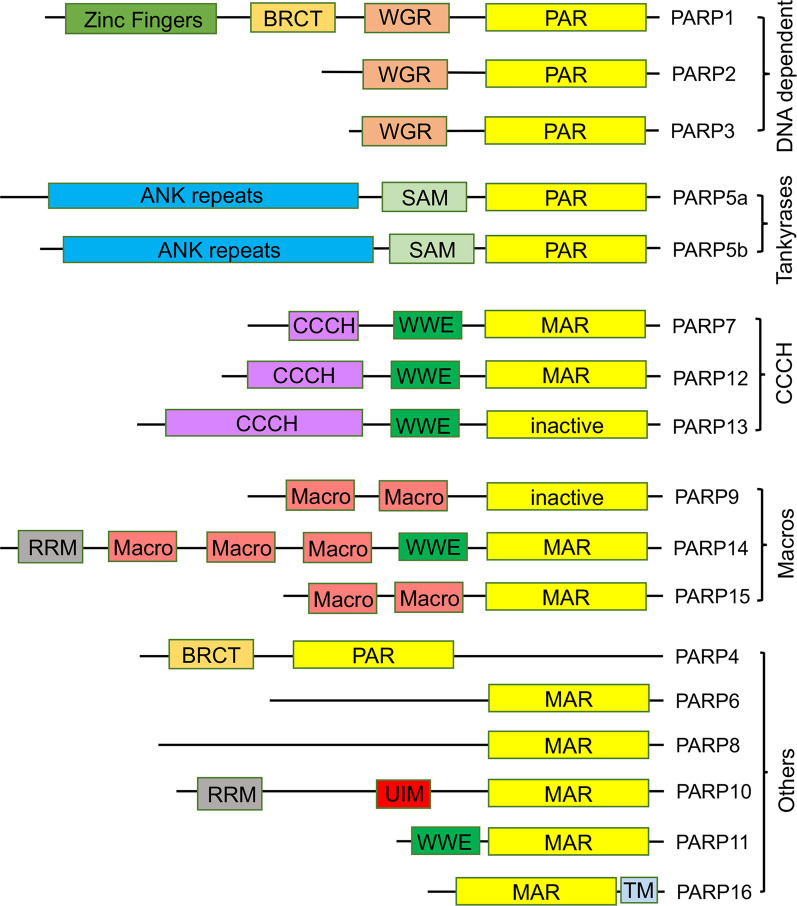
The domain organization of the five subfamilies of PARPs. The conserved PARP catalytic domain is indicated in yellow. The catalytic domain at the C terminus is conserved in all members and is required for NAD^+^ binding and PARylation activity. The zinc fingers domain in PARP1 is a DNA-binding site. The BRCA1 C terminus (BRCT), ankyrin repeat (ANK), sterile α-motif (SAM), and ubiquitin-interacting motif (UIM) domains are protein–protein interaction modules. The WGR, with conserved W, G and R residues, is a functionally unknown domain. The CCCH domain is a Cys–Cys–Cys–His zinc finger domain. The Macro and WWE (with conserved W, W and E residues) are ADP-ribose binding modules. The RNA recognition motif (RRM) is an RNA-binding motif. TM is a transmembrane domain.

The second category of NAD^+^-consuming enzymes include extracellular enzymes CD38 and BST1/CD157, as well as the intracellular sterile alpha and TIR motif containing 1 (SARM1) [23,24]. Although many reports show that they can convert NAD^+^ to cyclic ADP-ribose, the major *in vitro* activity is hydrolysis of NAD^+^ to ADP-ribose and nicotinamide (Figure 1, right) [25,26].

In recent years, it has been increasingly recognized that these NAD^+^-consuming enzymes play important roles in infection and inflammatory response. Below we will briefly review the roles of these NAD^+^-consuming enzymes in viral infection. We focus on the roles of PARPs because a large amount of data supports their vital role in fighting viral infections. Consistent with the role of NAD^+^-consuming enzymes in viral infection, the biosynthesis of NAD^+^ is also regulated during infection and inflammation, which will also be briefly summarized here.

The COVID-19 pandemic, which is still ongoing, reminds us that human society needs general antiviral strategies to fight newly emerging infectious diseases. While there are broad-spectrum antibiotics for treating bacterial infections, there are no general broad-spectrum antiviral strategies. Understanding how our immune system works to fight viral infections may help the development of general antiviral strategies for treating emerging pathogens. Thus, we hope that by understanding how NAD^+^-consuming enzymes help to restrict viruses and how viruses circumvent our immune defenses, we may come out with new broad-spectrum antiviral strategies.

## The roles of PARPs in viral infection

The antiviral roles of PARPs are supported by a large body of literature. In fact, one of the PARPs, PARP13, is also named zinc finger CCCH-type antiviral protein 1 (ZC3HAV1) or zinc finger antiviral protein (ZAP). Several other PARP family members are also reported to have antiviral function. Many PARPs are considered interferon-stimulated genes (ISGs), critical for innate immune response. Expression of PARP3, 4, 5a, 5b, and 7-was shown to be induced in cells infected by coronaviruses [22,27,28]. However, conflicting reports about the roles of PARPs in viral infection exist in the literature. Before we provide a more detailed description about the reported roles of PARPs in viral infection, we will explain a guiding hypothesis that helps us to synthesize the available information.

Our guiding hypothesis is that the PARP family members described below are in general evolved to help fight certain viral infections. However, certain viruses learned to circumvent the antiviral functions of PARPs or even take advantage of their presence to evade the host's immune response and establish successful infections. This is likely the reason why sometimes conflicting reports exist in the literature, with some reporting proviral roles while others reporting antiviral roles for PARPs. In other words, the antiviral roles of PARPs will likely depend on the types of viruses.

The complication could further come from the complexity of the immune system itself. The same biochemical function could serve either as anti-inflammation or pro-inflammation roles, depending on the context. For example, SARM1-catalyzed NAD^+^-degradation generally leads to cell death. If this happens in neurons, it leads to axon degeneration [29]. If SARM1 is activated in immune cells, such as cytotoxic T cells, it will likely promote T cell death [30], which could either promote viral infection or serve to prevent over-inflammation after the infection is taken care of. If this happens in virus-infected cells, the killing of the infected cells may help to limit the spread of the virus, which has been reported in plants [31,32]. Thus, the same biochemical function of SARM1 could lead to different immune outcomes depending on the context. This is an important point to keep in mind when considering the conflicting roles of NAD^+^-consuming enzymes in viral infection.

### PARP1

PARP1, which catalyzes PARylation, is the most extensively studied PARP member and inhibitors of PARP1 have been clinically used to treat cancers. The best-understood function of PARP1 is in DNA damage and repair [33]. However, many reports show that PARP1 is relevant for viral infections. The DNA repair function could indirectly contribute to fighting infection as reactive oxygen species (ROS) could induce DNA damage, thus having PARP1 active is important under increased oxidative stress caused by infections.

The role of PARP1 in viral infection depends on the virus (Table 1). For several DNA viruses, including adenovirus, Kaposi sarcoma-associated herpesvirus (KSHV), Epstein–Barr virus (EBV), and hepatitis B virus (HBV), PARP1 is reported to play a protective role. This protective role is mainly due to PARP1 interaction and modification of viral factors.

**Table 1:** Summary of PARP's roles in viral infections

Positive role for host	Ref.	Negative role for host	Ref.
Adeno associated virus (AAV) Kaposi sarcoma-associated herpesvirus (KSHV) Epstein–Barr virus (EBV) Hepatitis B virus (HBV) Human immunodeficiency virus 1 (HIV-1) Murine leukemia virus (MLV) Murine gammaherpesvirus 68 (MHV-68)	[36] [34] [37] [38] [46,52] [46] [39,40]	HIV-1 Influenza A virus (IAV)	[41–45] [50,51]
EBV	[55]	Herpes simplex virus (HSV-1)	[53]
Sindbis virus (SINV) Rubella virus Venezuelan equine encephalitis virus (VEEV)	[61] [61] [60]	Murine coronavirus (MHV)	[62]
Encephalomyocarditis virus (EMCV) IAV SINV Retrovirus VSV	[70] [71] [71] [71] [71]	N/A	
Avian influenza virus (AIV)	[72]	N/A	
Zika virus	[74]	VSV HSV-1	[73] [73]
Vesicular stomatitis virus (VSV) MHV-68 VEEV SINV Encephalomyocarditis virus (EMCV) Rift valley fever virus (RVFV) Chikungunya virus (CHIKV) Zika virus MHV	[78] [78] [80,82] [80] [80] [80] [80] [81] [62]	N/A	
Human T cell leukemia type 1 (HTLV-1) Japanese encephalitis (JE) virus Porcine reproductive and respiratory syndrome virus (PRRS) IAV HBV HIV-1 MHV-68 Alphavirus Newcastle disease virus (NDV) SARS-CoV-2	[85] [86] [87] [88,89] [89] [90] [91] [92] [98] [103]	N/A	
Human CoV-229E MHV	[62] [62]	N/A	

**Figure 3.**
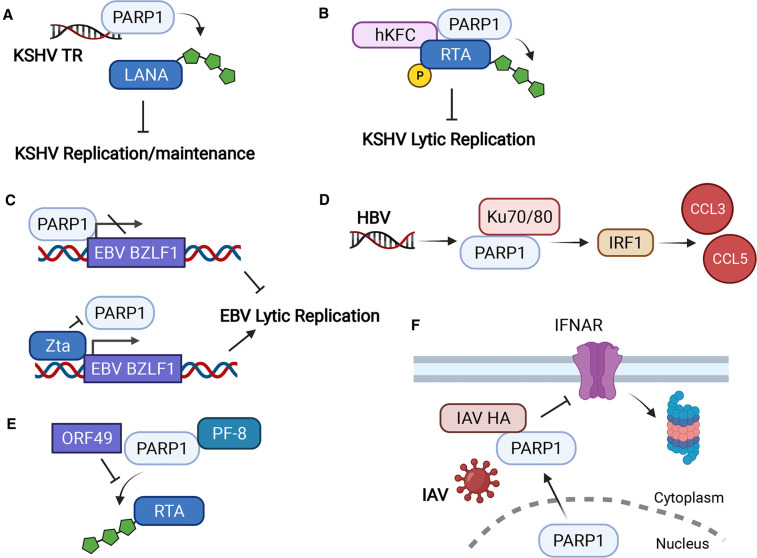
The functions of PARP1 in viral infection. (**A**) PARP1 binds to KSHV terminal repeats (TR) and catalyzes the ADP-ribosylation of LANA, reducing KSHV replication in the latency. (**B**) PARP1 and Ste20-like human kinase homologous to kinases from chicken (hKFC) together bind to and ADP-ribosylate/phosphorylate KSHV RTA protein, which suppresses RTA-mediated KSHV lytic reactivation. (**C**) PARP1 binds to EBV BZLF1 promoter to prevent its transcription, thus inhibiting EBV lytic reactivation; the viral protein Zta is sufficient to prevent PARP1 binding to the BZLF1 promoter, driving robust BZLF1 expression and lytic reactivation. (**D**) HBV DNA promotes Ku70/80 and PARP1 to activate interferon regulatory factors (IRFs), which induces chemokine CCL3 and CCL5 levels. (**E**) ORF49 or PF-8 binds to PARP1, and prevents PARP1 from interacting with RTA, which reduces PARylated RTA and enhances virus replication. (**F**) Upon IAV infection or overexpression of IAV HA, PARP1 interacts with HA and translocates from the nucleus to cytoplasm, further down-regulating interferon receptor IFNAR through proteasomal degradation. Created with BioRender.com.

For example, PARP1-catalyzed PARylation of latency-associated nuclear antigen (LANA) or transcription activator (RTA) protein reduces KSHV infection across two separate studies (Figure 3A,B) [34,35]. PARP1 PARylation combined with phosphorylation of RTA is proposed to reduce RTA binding to viral lytic promoters [31]. Similarly, in adenoviral infection, PARP1 binds adeno-associated virus (AAV) protein Rep to reduce viral genome integration [36].

In EBV infection, PARP1 itself binds a specific lytic promoter (BZLF1), blocking the transition from latency (Figure 3C) [37]. In response to HBV, PARP1 and Ku70/80 form a DNA-binding complex to promote interferon-stimulated genes (Figure 3D) [38]. This highlights a common theme where PARP1 plays several protective roles in the infection, both dependent and independent of its catalytic activity.

These DNA viruses have evolved to counter PARP1-mediated protection via multiple unique mechanisms. To counter PARP1 binding to the EBV lytic promoter, viral protein Zta out-competes PARP1 (Figure 3C). Both murine γ-herpesvirus 68 (MHV-68) and KSHV rely on RTA to promote lytic replication and utilize a processivity factor (PF-8) to bind to and promote degradation of PARP1 [39]. In addition, MHV-68 encodes an open reading frame, ORF49, that binds PARP1 and prevents it from interacting with RTA (Figure 3E) [40].

In contrast with the general protective role in DNA virus infection, the role of PARP1 in RNA virus infection is more complicated. For example, for the HIV-1 retrovirus, many reports indicate that PARP1 promotes HIV infection [41–45]. The pro-viral role is mostly mediated by PARP1's role in the transcription of integrated retroviruses [42,44,46,47]. However, some other studies showed that PARP1 is dispensable for HIV-1 integration, because retroviral replication still can proceed efficiently in PARP1-deficient mouse embryonic fibroblasts (MEFs) [48,49]. PARP1 also decreases interferon alpha/beta receptor (IFNAR) expression upon infection by influenza A virus (IAV) or overexpression of IAV hemagglutinin (HA). Mechanistically, HA interacts with PARP1 and promote its translocation from the nucleus to the cytoplasm, where PARP1 down-regulates IFNAR through proteasomal degradation (Figure 3F) [50]. Additionally, PARP1 can regulate IAV polymerase activity and affects IAV replication [51].

Some reports also indicate a protective role of PARP1 by suppressing HIV-1 gene transcription [46,52]. PARP1 competitively binds to the transactivation response element (TAR) RNA of HIV-1 with Tat/positive transcription elongation factor b (p-TEFb) complex, leading to p-TEFb displacement from HIV-1 RNA, suppressing Tat-mediated transcriptional elongation [52]. This PARP1-mediated retrovirus transcription inhibition is also efficient against murine leukemia virus (MLV), which is mediated by epigenetic mechanisms that involve DNA methylation and histone deacetylation but independent of the catalytic activity of PARP1 [46]. PARP1 can repress retroviruses prior to viral DNA integration by mechanisms involving histone deacetylases but not viral DNA integration and heterochromatin formation [47].

### PARP5a/b

**Figure 4.**
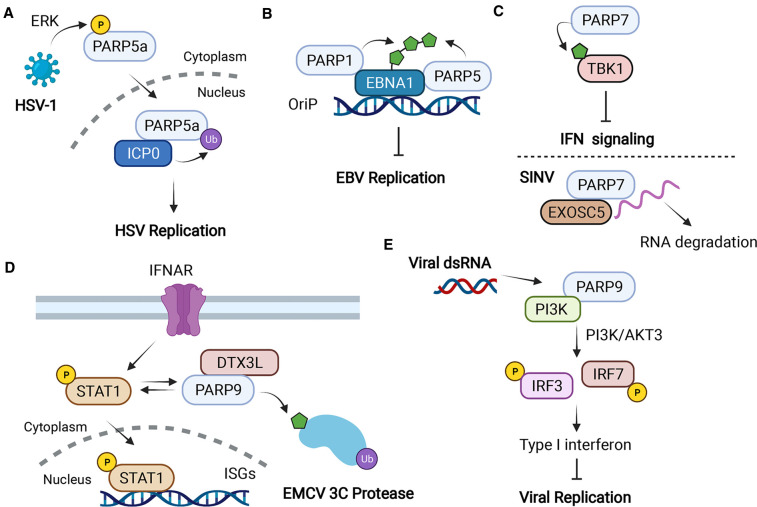
The functions of PARP5a/b, PARP7, and PARP9 in viral infection. (**A**) HSV-1 infection induces PARP5a phosphorylation via ERK and promotes its expression and translocation to the nucleus by interacting with ICP0, which results in proteasome-dependent degradation of PARP5a, enhancing HSV replication. (**B**) PARP1 or PARP5 binds and ADP-ribosylates EBNA1, which inhibits EBV replication. (**C**) PARP7 mono-ADP-ribosylates TBK1 and down-regulates type I interferon response to viral infection; Upon SINV infection, PARP7 binds viral RNA and EXOSC5 (an exosome component) for RNA degradation. (**D**) IFN-induced overexpression of STAT1 up-regulates of PARP9 and DTX3L. In turn, PARP9/DTX3L complex binds and promotes STAT1 phosphorylation, nuclear localization, and increases ISGs levels, and triggers degradation of EMCV 3C proteases; (**E**) PARP9 recognizes viral dsRNA from RNA viruses and employs PI3K/AKT3 pathway to phosphorylate IRF3 and IRF7 for inducing type I IFN, which inhibits the RNA virus infection. Created with BioRender.com.

PARP5a and PARP5b, also known as Tankyrases 1 and 2 (TRF1-interacting, ankyrin-related ADP-ribose polymerases) are required for Herpes simplex virus (HSV-1) infection. During HSV-1 infection, PARP5a is phosphorylated via extracellular signal-regulated kinase (ERK), translocates to the nucleus, and co-localizes with infected cell protein 0 (ICP0), an immediate early viral protein that functions as an E3 ubiquitin ligase (Figure 4A). This leads to proteasome-dependent degradation of PARP5a at the late stage of infection. Knockdown of both PARP5a and PARP5b, or inhibition of their catalytic activity using XAV-939 results in the reduction in viral protein expression and replication [53].

In contrast, in EBV, PARP5 has protective roles. EBV can persist in latency and replicate its genome once per cell division cycle. This replication is dependent on the viral protein EBNA1 binding to its double-stranded DNA at the origin of plasmid (OriP) replication site [54,55]. The EBV OriP structure consists of the family repeats (FR) and a dyad symmetry region. In addition to four EBNA1 binding sites, the dyad symmetry region contains sites (TTAGGGTTA) that bind telomere repeat binding factors TRF1 and TRF2. The factors are important for OriP plasmid maintenance and DNA replication [54]. One study reported that TRF1 binding to telomeric repeats can be regulated by PARP5 [56]. Furthermore, PARP5a/b and TRF2 were identified to interact with the dyad symmetry region in an EBNA1-dependent manner. PARP5 down-regulates OriP replication and plasmid maintenance through PARP activity (Figure 4B) [54,55]. PARP1 can also bind and PARylate EBNA1; its binding to TRF2 induces dyad symmetry structure changes, which impairs EBNA1 DNA binding and functional recruitment of origin recognition complex [57].

### PARP7

PARP7, or 2,3,7,8-tetrachlorodibenzo-p-dioxin (TCDD)-inducible PARP (TiPARP), is one of the CCCH zinc finger domains containing PARPs. Unlike PARP1 or PARP5a/b, PARP7 is a mono-ADP-ribosyl transferase. It can bind DNA or RNA and is regulated by aryl hydrocarbon receptor (AHR) [58,59]. PARP7's various functions are tied to its inducible nature, as it can act as a stress response under conditions such as hypoxia [15] or viral infection [60–62]. PARP7 can be induced by a growing list of transcription factors including androgen receptor (AR) [63], estrogen receptor (ER) [64], AHR [59], and HIF-1α [15]. PARP7 has auto-mono-ADP-ribosylation activity that promotes its own degradation resulting in a half-life estimated to be only 4.5 min [15,65,66]. The unique characteristics of PARP7 gives the ability to quickly modulate a cellular response to viral infection.

The role of PARP7 in viral infection is variable and system dependent. For example, PARP7 is up-regulated after mouse hepatitis virus (MHV) infection in bone-marrow derived macrophages (BMDMs). Knockdown of PARP7 slightly reduces the viral RNA production, which suggests a pro-viral function [62]. AHR signaling down-regulates type I interferon (IFN-1) response to multiple virus types [67]. In this setting, PARP7 mono-ADP ribosylates tank binding kinase (TBK1), a well-known modulator of type I IFN signaling. This ribosylation down-regulates type I IFN response to viral infection by reducing TBK1 S172 phosphorylation (Figure 4C). A recent study found that PARP7 promotes degradation of AHR itself and reduces *Ifnb1* expression in response to 5′-triphosphate-RNA [68]. Taken together, AHR signaling and PARP7 can be viewed as a ‘turn-off' mechanism to avoid deleterious effects of excessive interferon signaling. PARP7 turns off type I IFN response while simultaneously down-regulating the transcription factor (AHR) responsible for this turn-off.

In contrast, PARP7 is reported to have antiviral activity in the *Togaviridae* virus family (positive-strand RNA viruses). PARP7 specifically degrades genomic RNA of Sindbis (SINV) and Rubella viruses in an exosome-dependent manner [57]. Knockdown of PARP7 in U373 human astrocyte cells or knockout of PARP7 in mice leads to enhanced replication of SINV and Rubella virus replication, but it fails to increase replication of other RNA viruses. Thus, the antiviral effect of PARP7 is virus-dependent.

Mechanistically, CCCH-type zinc finger domain-containing proteins regulate RNA degradation and protein translation [69]. PARP7 binds to SINV RNA via its N-terminal CCCH-type zinc-finger domain and induces RNA degradation by recruiting exosome complex component 5 (EXOSC5) (Figure 4C) [61]. In a different study, PARP7, along with PARP10 and PARP12 inhibits a venezuelan equine encephalitis virus (VEEV) replication model. All three PARPs impact viral replication by blocking protein translation [60], indicating another beneficial role for PARP7 in fighting *Togaviridae* family viruses.

### PARP9

PARP9 is catalytically inactive but still mediates antiviral immune response. One study utilized a hyper-efficient STAT1 double-mutant transgenic mouse model to uncover an important role of a PARP9 and Deltex E3 ubiquitin ligase 3L (DTX3L) complex [70]. The STAT1-mutant mice are significantly better at reducing replication of multiple viruses, including encephalomyocarditis virus (EMCV), IAV, and sindbis virus (SINV) but lose efficiency with knockdown of either PARP9 or DTX3L. It is interesting these effects are independent of the PAR-binding function of the PARP9 macrodomains but require the ubiquitination function of the DTX3L-RING domain. The complex interacts with STAT1 and utilizes its E3 ligase activity on histone H2BJ to promote ISG expression (Figure 4D). The complex can also promote degradation of viral EMCV 3C protease. This study highlights PARP9's role in viral response but it is unclear if PARP9 plays a predominant role for wild-type STAT1.

Other reports also connect PARP9 to IFN signaling. PARP9 was recently discovered to be a non-canonical sensor for RNA viruses to promote type I IFN production via the phosphoinositide 3-kinase (PI3K)/AKT3 pathway (Figure 4E) [71]. Knockdown or deletion of PARP9 in dendritic cells, macrophages, or mice inhibits type I IFN production in response to double strand RNA (dsRNA) stimulation or RNA virus infection, including retrovirus, VSV, and IAV. Mechanistically, PARP9 uses its macrodomains for recognizing viral dsRNA, and interacts with PI3K p85 for activating downstream PI3K/AKT3 pathway, independent of mitochondrial antiviral-signaling (MAVS), limiting the RNA virus infection [71].

### PARP10

**Figure 5.**
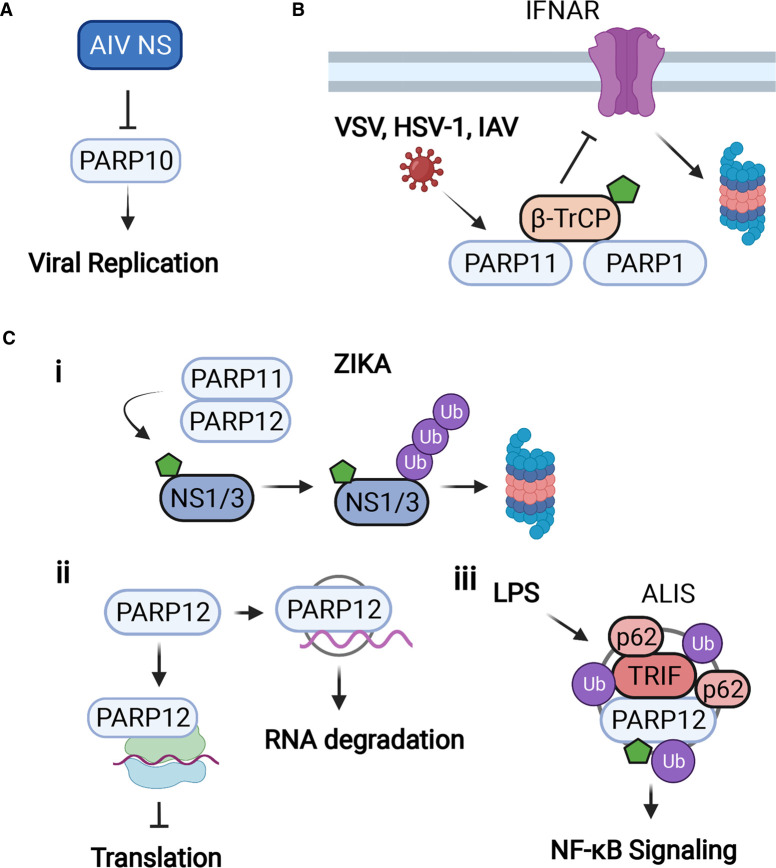
The functions of PARP10, PARP11, and PARP12 in viral infection. (**A**) AIV NS binds to and down-regulates PARP10, which enhances virus replication. (**B**) PARP11 expression is up-regulated during VSV, HSV-1, and IAV virus infections, and PARP11, like PARP1, promotes VSV and HSV-1 infection by mono-ADP-ribosylating β-TrCP, leading to the ubiquitination and degradation of IFNAR. (**C**) (**i**) PARP12 expression leads to ADP-ribosylation of Zika proteins NS1 and NS3, leading to their proteasome-mediated degradation, and PARP11 also interacts with PARP12 to enhance Zika virus NS1 and NS3 degradation but independent of its PARP activity; (**ii**) PARP12 both blocks viral protein translation and binds viral RNA for degradation; (**iii**) Upon LPS treatment, ALIS structures are positive for PARP12, p62, ubiquitin, and TRIF forms, which regulates NF-κB signaling. Created with BioRender.com.

PARP10 is one of the interferon-stimulated PARPs with MARylation activity. Knockdown of PARP10 increased avian influenza virus (AIV) replication while overexpression of PARP10 reduced AIV [72]. AIV nonstructural protein interacts with PARP10, promotes PARP10 nuclear localization, and reduces endogenous PARP10 expression (Figure 5A), highlighting an interesting countermeasure evolved by AIV. However, this study lacks data in mouse models to more rigorously demonstrate PARP10's antiviral role.

### PARP11

PARP11 is the second smallest PARP, with only a single WWE domain and a MARylation catalytic domain. It is also highly up-regulated by IFN [62]. PARP11 promotes VSV and HSV-1 infection by inhibiting the interferon response [73]. Like PARP1, PARP11 can inhibit IFN signaling by mono-ADP-ribosylating the E3 ubiquitin ligase β-transducin repeat-containing protein (β-TrCP), leading to the ubiquitination and degradation of IFNAR (Figure 5B). PARP11 overexpression restricts the ubiquitin-proteasome degradation of β-TrCP. PARP11 expression is significantly up-regulated during virus infection, including VSV, HSV-1 and IAV. PARP11 knockdown or a pan-PARP inhibitor (rucaparib) treatment limits the replication of VSV and HSV-1. Importantly, the inhibitory effects of rucaparib on viral infection and enhanced ISG expression are largely diminished by PARP11 knockdown. Thus, PARP11 could be a potent regulator of the IFN signaling pathway and antiviral activity [73].

In contrast, PARP11 is identified as an anti-Zika virus ISG. It interacts with PARP12 via its WWE domain to enhance Zika virus NS1 and NS3 protein degradation (Figure 5C i) [74]. PARP11 is up-regulated in WT but not IFNAR1^−/−^ cells in response to IFNα/β stimulation and Zika virus infection. Zika virus replication is only suppressed in cells expressing PARP11. Interestingly, PARP11 deletion mutants either lacking the WWE domain or PARP domain do not affect NS1 and NS3 degradation [74].

### PARP12

PARP12 is a mono-ADP-ribosyltransferase with four N-terminal CCCH-type zinc-finger domains, a single WWE domain at its center, and a C-terminal PARP catalytic domain. The zinc-finger domains bind both viral and cytoplasmic RNAs and play important roles in immune cells such as macrophages [69,75–77]. PARP12 is also one of many ISGs [78–80]. The antiviral role of PARP12 was first described in an overexpression screen, where it mildly inhibited the replication of both VSV and MHV-68 [78]. PARP12 was differentially expressed in cells that cleared VEEV replication compared with those that were persistently infected [80]. Further analysis showed that expression of PARP12L, but not PARP12S, restricts VEEV replication, as well as several other viruses including SINV, EMCV, VSV, rift valley fever virus (RVFV), and chikungunya virus. PARP12 also has a role in the restriction of coronavirus replication. PARP12 can restrict MHV replication lacking the ADP-ribosyl-hydrolase (ARH) activity of the coronavirus macrodomain in bone marrow derived macrophage cells [62]. Interestingly, PARP12 was identified in a screen for ISGs that inhibit Zika virus [81]. Using both knockout and overexpression, the authors showed that PARP12 was both necessary and sufficient for the inhibition of Zika virus replication. PARP12 across many virus types and in many systems appears to have a robust protective effect.

The antiviral effect of PARP12 can be achieved through several mechanisms. It can block translation, bind viral RNA at stress granules to promote its degradation [22,80,82], and directly modify viral proteins [28,62,81]. For example, Zika nonstructural proteins NS1 and NS3 are poly-ADP-ribosylated dependent on PARP12 catalytic activity (Figure 5C i). This modification promotes K48-ubiquitination and degradation of these viral components. In contrast with a recent mechanism proposed for PARP7-mediated degradation of HIF-1α, neither PARP12 WWE domain nor the zinc finger domains are required for this model [15,81]. Notably, the authors further hypothesize that PARP12 mediated MARylation is rate-limiting step prior to the observed PARylated Zika proteins. Although only NS1 and NS3 are reported thus far, it is plausible this mechanism exists for other viral proteins.

In a VEEV infection model, PARP12 (along with PARP7 as previously discussed) is the most effective at reducing viral replication through host translation inhibition [80,82]. Using polysome profiling and mass spectrometry, this study shows that PARP12 exists in two types of complexes: bound to ribosomes and bound to RNA. PARP12 shows cytosolic punctate localization presumably at RNA centers [82], which is dependent on its catalytic activity. PARP12 interaction with RNA is consistent with an RNA interactome of SARS-CoV-2, which identified PARP12 as one of the hits [83]. However, whether the cytosolic stress granule localization contributes to translation inhibition, viral RNA degradation, or multiple mechanisms is not fully understood (Figure 5C ii).

It is documented that endogenous PARP12 in HeLa cells can re-localize into various ‘bodies' in response to stress [84]. Endogenous PARP12 forms cytosolic bodies after IFN-β or lipopolysaccharide (LPS) treatment termed ‘aggresomes-like structures' (ALIS) in RAW264.7 immortalized macrophages [82] (Figure 5C iii). The data suggests these structures are sites rich in immune-related proteins such as NF-κB that may be regulated by PARP12. Overall, PARP12 bodies have several reported roles related to antiviral defense ranging from binding RNA to sites of immune signaling. Understanding the detailed molecular mechanism will help explain PARP12's critical role in antiviral defense.

### PARP13

PARP13, better known as zinc finger antiviral protein (ZAP), is well characterized as an antiviral defense mechanism. Though catalytically inactive, PARP13 has broad-spectrum antiviral activity against human T cell leukemia virus type 1 (HTLV1) [85], Japanese encephalitis virus [86], porcine reproductive and respiratory syndrome (PRRS) virus [87], IAV [88], HBV [89], HIV-1 [90], MHV-68 [91], alphavirus [92,93], and L1 retrotransposition [94]. Given its strong and broad antiviral effects, it is not surprising that several viruses have evolved to counteract PARP13 by degrading PARP13 protein or mRNA [95,96].

In response to both RNA and DNA viruses, ZAP inhibits translation and promotes viral RNA degradation across experimental settings [76,97]. There are four known ZAP isoforms: ZAP extra-long (XL), ZAP-long (L), ZAP-medium (M), and ZAP-short (S). Only ZAPL and ZAPS are highly expressed; thus, their functions are the best characterized. ZAPL contains a PARP domain that is missing key catalytic residues, while ZAPS does not contain the PARP domain. All ZAP isoforms contain two RNA binding domains (RBD), five different zinc finger domains, and two WWE domains. A recent review on ZAP specifically highlights its roles in targeting many different viral families from *Coronaviridae* to *Togaviridae* [97].

ZAPL and ZAPS have remarkably different characteristics. ZAPL is constitutively expressed and acts as an intrinsic antiviral effector, while ZAPS, upon induction by interferon, is a potent stimulator of signaling mediated by the RNA helicase RIG-I during antiviral responses. Mechanistically, ZAPS interaction promotes RIG-I oligomerization, ATPase activity, and induction of interferon response in response to IAV or Newcastle disease virus [98].

**Figure 6.**
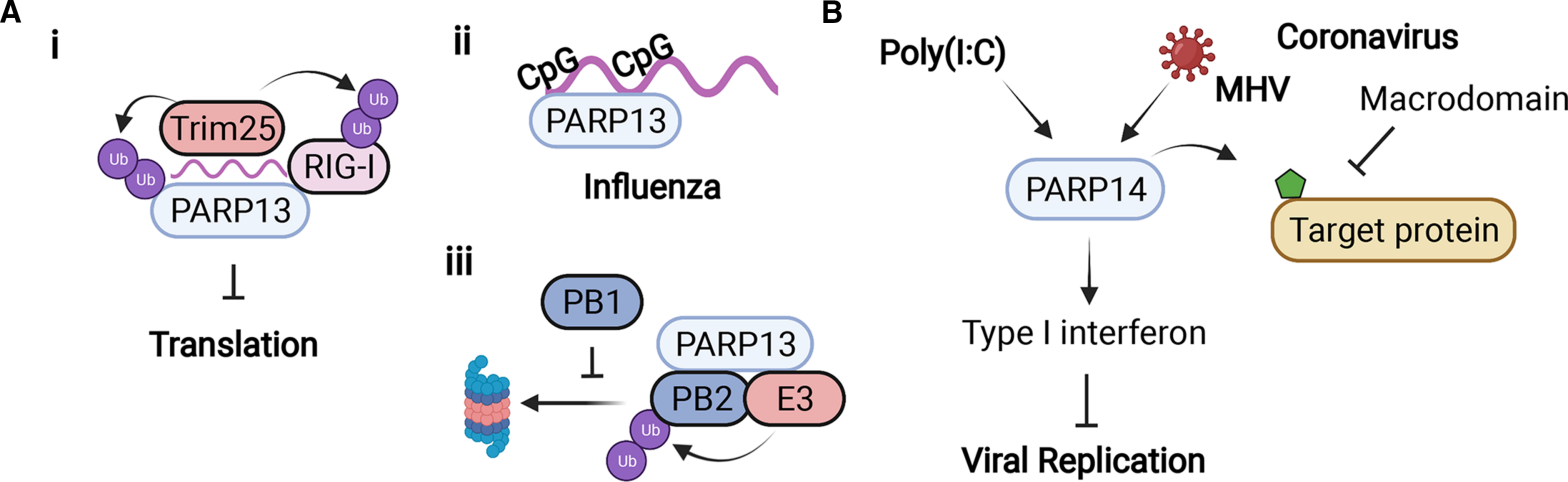
The antiviral functions of PARP13 and PARP14. (**A**) (**i**) PARP13 co-factor Trim25 ubiquinates both PARP13 and RIG-I to promote antiviral activity; (**ii**) PARP13 has high affinity for RNA viruses rich in CpG dinucleotides; (**iii**) PARP13 promotes ubiquitination, and degradation of influenza protein PB2 (and also PA, not shown), which is blocked by competition from influenza protein PB1 for PARP13 binding. (**B**) PARP14 is required for enhancing IFN production in response to CoV infection (MHV) or poly(I:C) treatment; meanwhile, coronavirus macrodomain prevents the antiviral effect of PARP14 by removing ADP-ribose from target protein. Created with BioRender.com.

ZAP can help to fight viral infection by binding to viral RNA, which promotes viral RNA degradation and inhibition of mRNA translation. It was originally documented that ZAP binds and destroys specific viral mRNAs as early as 2002 [75,99]. First determined in HIV-1, the ZAP RBDs have a high affinity towards CpG dinucleotides (Figure 6A ii) [97,100]. The field has coined the term ZAP-response element (ZPE) to describe a region of viral coding with high CpG content. Vertebrate genomes have evolved to robustly suppress CpG content and thus lower the frequency of ZPEs. Low CpG-containing viruses, such as HIV-1, are unaffected by ZAP, whereas unnatural additions of CpG to their genomes leads to susceptibility to ZAP expression [100]. It has been shown that ZAP selectively targets multiple spliced viral mRNAs for degradation [90] and inhibits the translation of viral mRNA [101]. The stress granule localization of ZAP is correlated with its antiviral activity [102].

Despite relatively low CpG rates, SARS-CoV-2 viral infection is restricted by ZAPL and ZAPS [103]. This effect is potentiated by the addition of interferons which induces more ZAP, among other PARP family members [62,103]. ZAP, along with Trim25 and PARP12 (discussed in the PARP12 section and below), directly bind SARS-CoV-2 RNA [83]. To counter ZAP targeting, SARS-CoV-2 shows evidence of CpG suppression over the course of a 5-month study [104]. Furthermore, phylogenetic mutation analysis suggests that C and G mutations are reflective of SARS-CoV-2's attempts to counter host defense systems such as ZAP targeting [105].

Like other PARP family members, ZAP plays additional roles in degradation of viral RNA or proteins. Specifically, ZAP recruits host cofactors, such as E3 ubiquitin ligase, Trim25, to target and destroy viral DNA or RNA. This enzyme can ubiquitinate ZAP itself, bind RNA, and improve ZAP antiviral activity across multiple studies (Figure 6A i) [97,103]. After binding, ZAP-mediated degradation proceeds via complicated mechanisms involving exoribonuclease complexes and RNA helicases to unwrap and degrade RNA [97].

ZAPL can also exert antiviral activity by promoting the degradation of viral proteins. For IAV, ZAPL binds the viral PB2 and PA polymerase proteins, leading to their proteasomal degradation (Figure 6A iii). After the PB2 and PA proteins are ADP-ribosylated, they are associated with the region of ZAPL that includes both the PARP domain and the adjacent WWE domain that is known to bind ADP-ribose. These complex proteins are then ubiquitinated, followed by proteasomal degradation. This antiviral activity is counteracted by the viral PB1 polymerase protein, which binds close to the PARP domain and causes PB2 and PA to dissociate from ZAPL and escape degradation. This could explain why ZAPL only moderately inhibits IAV replication. Eliminating PB1 binding to ZAPL would substantially increase the inhibition of IAV replication, so that the PB1 interface with ZAPL is a potential target for antiviral development [88].

ZAP also has additional antiviral roles beyond regulating viral RNA or protein degradation. For example, ZAP can directly bind host RNAs and specifically regulate their translation to tune the immune response. Specifically, ZAP can decrease TRAILR4 translation and can modulate TRAIL-regulated apoptosis [106]. Some reports indicate ZAP may either repress or promote IFN response gene translation via different mechanisms. More interestingly, there are reports that under some viral infections such as HIV-1, RNAi activity is turned down directly after infection and this promotes IFN response. This is because ZAP promoted ADP-ribosylation of core RNAi machinery components [107].

### PARP14

PARP14 plays a crucial role in many immune responses, such as macrophage activation [108] and accumulation of type I IFN–inducible proteins [109]. PARP14 contains multiple macrodomains, which can bind to ADP-ribose. Residues in the ADP-ribose binding pocket have been identified to be essential for macrodomain activity [110]. Several viruses, including those from *Coronaviridae*, *Togaviridae*, and *Hepeviridae,* also encode a macrodomain that binds to and counters cellular ADP-ribosylation to combat the immune response [111–113]. SARS-CoV and human CoV 229E with macrodomain-mutant showed increased sensitivity to type I IFN treatment, implying that the CoV macrodomain counters antiviral activities of ISGs [114]. One study demonstrated that PARP14 shows antiviral function in primary macrophage cells during MHV infection, and in human A549 cells with poly(I:C) treatment (Figure 6B) [62]. Specifically, knockdown of PARP12 or PARP14 leads to increased replication of MHV with a mutant-macrodomain (N1347A) but has little impact on wild-type virus in bone marrow-derived macrophages (BMDMs). By using a PARP14-specific inhibitor 8K, along with PARP14^−/−^ BMDMs, A549 and normal human dermal fibroblast cells, the authors further confirm that PARP14 is necessary to enhance the type I IFN induction following coronavirus infection or poly(I:C) stimulation [62]. Consistent with this finding, another study discovered sequence similarity between the ADP-ribose-binding domain of SARS-CoV-2 and PARP14. This suggests coronaviruses coevolve with ADP-ribosylation enzymes to counter the ADP-ribosylation activity of PARPs and their antiviral immune response [115].

## The roles of other NAD^+^-consuming enzymes in viral infection

### The role of sirtuins

There are seven sirtuins, SIRT1-7, in humans. They regulate diverse functions, including transcription, genome stability, metabolism, and cell signaling via deacylating various substrate proteins [116,117]. Although the roles of sirtuins in viral infection have not been investigated extensively and the literature does not provide a unifying picture, some evidence points to a protective role of sirtuins in fighting infection. One report showed that all seven sirtuins have broad-range antiviral properties and knocking down any of them promotes the production of virus progeny in infected human cells for many different viruses [118]. The mechanisms behind this promotion are unknown. Protective roles of specific sirtuins in combating infection have been reported as well. SIRT1 promotes the differentiation of Th17 cells [119], which is critical for immune response against microbial infections. Disruption of SIRT1 also increases HPV16 E1-E2 replication [120] and enterovirus 71 replication [121]. SIRT6 promotes TNFα secretion [6,122], dendritic cell differentiation [123], and SIRT6 knockdown is reported to promote viral growth of cytomegalovirus in both fibroblasts and macrophages [124].

In support of a protective role, several well-known properties of sirtuins could provide beneficial effects to fight infections. One of the most established functions of SIRT1 is to promote autophagy and lysosomal function [125,126]. Autophagy and lysosomes are critical for fighting various infections [127]. One well-known function of SIRT3 and SIRT5 is to increase cellular NADPH production by regulating various metabolic enzymes that produce NADPH, such as IDH2 [128,129]. NADPH is required to generate ROS through NADPH oxidases [130]. ROS is a major chemical defense mechanism of our immune system towards microbial infection [130]. Thus, during a severe infection, ROS production increases significantly and SIRT3 and SIRT5 could promote ROS by increasing NADPH production. ROS will also damage normal tissues and thus must be well controlled. Paradoxically, NADPH is also required to repair the oxidative damage caused by ROS to cellular proteins. Thus, sirtuins could also be important for controlling the tissue damage caused by infection.

### The role of CD38 and SARM1

The NAD^+^ glycohydrolase CD38 is also important for controlling infections. It is a type I membrane protein with the active site on the extracellular surface of cells (or the luminal side of intracellular organelles). Infection by several viruses, including HIV-1, and by bacteria, is known to trigger CD38 expression [131–133]. In fact, in many cases, CD38 has been used as a marker for immune cell activation [134]. CD38 knockout mice have been shown to have defects in fighting bacterial infection [135–137]. Similarly, the sister protein of CD38, CD157, which is also an ecto NAD^+^ glycohydrolase, has been shown to be important for immune responses [138].

The intracellular NAD^+^ glycohydrolase, SARM1 [29], is a negative regulator of TLR-mediated NF-κB activation [139,140]. The well-known role of SARM1 in promoting axon degeneration has recently been linked to innate antiviral immune response [141–143]. During la crosse virus (LACV) infection, SARM1 is up-regulated and translocates to the mitochondria where it interacts with MAVS. This eventually leads to neuronal cell death via mitochondrial damage and oxidative stress. Additionally, SARM1 regulates cell survival and cytokine release following inflammasome activation [144]. It is interesting that the same enzymatic activity can either promote or suppress immune responses, depending on the context. SARM1 activation in immune cells, such as cytotoxic T cells, will promote T cell death [30], which could either promote viral infection or serve to prevent over-inflammation after the infection is taken care of. SARM1 activation in virus-infected cells will lead to killing of the infected cells, which may help to limit the spread of the virus [31,32]. Thus, the same biochemical function of SARM1 could lead to different immune outcomes depending on the context, an important point to keep in mind when considering the often conflicting roles of NAD^+^-consuming enzymes in viral infection.

## The regulation of NAD^+^ biosynthesis during infections

If NAD^+^-consuming enzymes, especially PARPs, are important in fighting infections, it would be expected that NAD^+^ biosynthesis would also be regulated during infections. This is indeed the case.

In mammals, there are two NAD^+^ biosynthesis pathways, the salvage pathway and the de novo pathway (Figure 1, left) [145]. In the salvage pathway, nicotinate is converted to nicotinate mononucleotide (NaMN) by nicotinate phosphoribosyltransferase (NAPRT), which is then adenylated to generate nicotinate adenine dinucleotide (NaAD^+^) by NaMN adenyltransferase (NMNAT). NaAD^+^ is then converted to NAD^+^ via NAD synthetase. Nicotinamide can be similarly converted to NAD^+^. Nicotinamide riboside, which is present in milk, can also be phosphorylated by its kinases to produce NMN and feed into the salvage pathway [146].

The de novo pathway starts from the amino acid tryptophan. Three heme-dependent enzymes (IDO1, IDO2, and TDO2) can oxidize tryptophan to N-formylkynurenine, which is further processed by four other enzymes to produce nicotinate mononucleotide (NaMN) and feed into the salvage pathway (Figure 1).

Given the above generalization that NAD^+^-consuming enzymes are important for fighting infection, under a severe infection, NAD^+^ will be substantially consumed by these enzymes and thus cells must increase the production of NAD^+^. Indeed, key enzymes in both the salvage and de novo pathways are known to be up-regulated by infections. NAMPT, the rate-limiting enzyme in the salvage pathway, is up-regulated by several different viruses, including HIV and Zika virus [147,148]. Searching publicly available microarray and next-generation sequencing data revealed that NAMPT transcription is significantly up-regulated during many immune-related processes, such as myeloid differentiation [149], lipopolysaccharide (LPS) treatment [150,151], and T cell activation [152]. This transcriptional data is consistent with the idea that under infections, NAD^+^ biosynthesis needs to be increased. One report suggests that NAMPT transcription is controlled by STAT1, which is activated by type I interferons [124]. A recent report showed that NAMPT is up-regulated by SARS-CoV-2 infection in A549 cells, which is also consistent with these earlier findings. However, two other enzymes, NADSYN and QPRT, were down-regulated, suggesting that the regulation of NAD^+^ biosynthesis enzymes is more complicated than we currently understand.

The gatekeepers of the de novo pathway, IDO1/IDO2/TDO2, are also heavily up-regulated during immune activation. IDO1 level and reaction product are up-regulated by human/simian immunodeficiency virus infection [153]. IDO1 induction is through interferon-STAT and is a well-known interferon-induced gene [154]. DNA microarray or next-generation sequencing data also showed that IDO2 and TDO2 are up-regulated by infection or immune activation [151,155–159]. IDO1 is reported to help restrict viral infection [160]. IDO1/IDO2/TDO2 have been well documented to have strong immune modulatory functions [161]. It has been proposed that the intimate connection between IDO1 and immune response is due to the immune modulatory roles of the intermediary metabolites in the de novo NAD^+^ pathway [161] or due to the depletion of tryptophan [162], which restricts T cell proliferation. However, given that NAD^+^-consuming enzymes could help fight infection, an alternative explanation for the up-regulation of IDO1/IDO2/TDO2 is to promote NAD^+^ biosynthesis. Consistent with this view, the enzyme in the last step of the de novo pathway, quinolinate phosphoribosyltransferase (QPRT), is reported to be an antiviral host factor against Hepatitis C infection [163]. However, QPRT is down-regulated by SARS-CoV-2 infection in A549 cells [28]. Thus, the exact role of this NAD^+^ biosynthesis pathway in fighting viral infection needs to be further investigated. It is possible that viruses have learned to suppress NAD^+^ biosynthesis to evade the host immune response.

The roles of NAD^+^-consuming enzymes like PARPs and dependence on NAD^+^ could also potentially explain the function of NAD^+^ glycohydrolases, CD38 and CD157. These two ecto-enzymes, with their active sites in the extracellular space, serve to mainly degrade extracellular NAD^+^, which is likely released from infected cells sacrificed by cytotoxic T cells and natural killer cells. The degradation products, nicotinamide and ADP-ribose (which can be further degraded to AMP and ribose-5’-phosphate), can be taken up by nearby live immune cells and used to synthesize more NAD^+^. This will in turn help to boost NAD^+^ synthesis and fight the infection. This hypothesis should be tested in future research.

## Viruses counteract NAD-mediated host defense

The above discussion on PARPs and sirtuins, regulation of NAD^+^ biosynthesis, and NAD^+^ degrading enzymes provides a unifying hypothesis/model that connects each of them to fighting infections. This model is further supported by findings on the viruses. The genome of SARS-CoV-2 encodes a protein, NSP3, which contains a macrodomain. Macrodomains are evolutionarily conserved and are present in many different virus families, including the *Coronaviridae*, *Togaviridae*, *Matonaviridae*, and *Hepeviridae* families [110]. Macrodomains are known to have two functions. They specifically bind to ADP-ribosylated proteins and thus mediate many of the biological effects of protein ADP-ribosylation [164–166]. In fact, many of the human PARPs proteins that are important for fighting infections contain macrodomains [18]. Therefore, after they modify themselves and substrate proteins, they can also bind to the modified proteins via the macrodomains, which somehow help to achieve their immune-modulatory function. Some macrodomains also have enzymatic activities. They can hydrolyze ADP-ribosyl groups off the modified proteins or small molecules [167–170]. Eight recent reports showed that the macrodomains from different viruses (including coronaviruses) can hydrolyze ADP-ribosyl groups installed by host PARP proteins, thus counteracting the antiviral activities of PARPs. Additionally, the catalytic activity of the viral macrodomain is important for virulence [62,111,112,171–176]. The presence of viral macrodomains to reverse the host PARPs-mediated ADP-ribosylation and the importance of the viral macrodomains for virulence is a strong testament to the importance of PARPs and NAD^+^ in immune responses (Figure 6).

## Implications for treating viral infections

**Figure 7.**
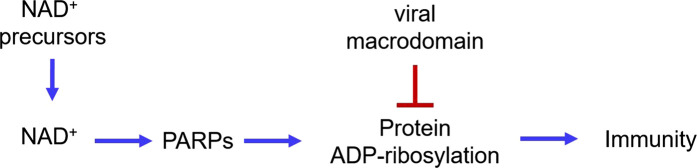
Scheme showing that NAD^+^ and PARPs promote immune responses to fight infections while viral macrodomains inhibit this immune defense mechanism. Promoting NAD^+^ and PARPs-mediated defense mechanisms, as well as inhibiting viral macrodomains, could potentially help fight viral infections.

The above analysis suggests that host cells deploy NAD^+^-consuming enzymes to fight infection and concurrently boost NAD^+^ biosynthesis to ensure the NAD^+^ supply. However, viruses, including SARS-CoV-2, sabotage this defense mechanism by reversing the effect of PARPs with macrodomains (Figure 7). As alluded to for PARP14, one may suggest the development of small molecule inhibitors for the macrodomains as antiviral agents. Such inhibitors must be selective for viral macrodomains because if they also bind to host macrodomains, they may interfere with the host's defense mechanism.

Using NAD^+^ biosynthesis precursors (nicotinate, nicotinamide, NR, NMN, and tryptophan) to boost host NAD^+^ biosynthesis could be another potential way to help treat viral infections. This will help to ensure NAD^+^ supply and thus fortify our natural defense mechanism (Figure 7). Given the tug-of-war between the virus and the host, supplying NAD^+^ biosynthesis precursors might help to tip the balance toward clearing the infection. Nicotinate or nicotinamide are already in commonly used vitamin supplements and thus are easily available. In recent years, many researchers and companies are pushing the idea of using NR or NMN to increase NAD+ and obtain health benefits [177–182]. These molecules are commercially available due to these efforts. It is still too early to conclude whether NR or NMN would bring health benefits under normal conditions [181–184]. However, given the above analysis, it would be interesting to test whether these NAD^+^ precursors would be beneficial under viral infections.

A counter argument for any beneficial effects of NAD^+^ precursors is that our immune system is already optimized to produce the NAD^+^ needed, and thus supplying extra NAD^+^ precursors may not have much effect. Alternatively, successful pathogens may already have developed strategies to evade the effects of host's NAD^+^-consuming enzymes, and thus increasing the NAD^+^ supply may not be very useful. A recent study showed that this might be the case. Supplying NAD^+^ precursors only helped to fight infections of MHV with an inactive macrodomain, but not wild-type MHV [185].

Another potential antiviral strategy is to use small molecules that can induce the expression of PARPs and thus enhance the antiviral immune response. This is possible as the expression of many PARPs is inducible. While interferons can obviously achieve this, we believe using small molecules to induce PARPs are more ideal. One of the most interesting examples is PARP7. It can be induced by small molecules like TCDD and estradiol, via AHR and ER nuclear receptors [15]. More and safer small molecules that can induce the expression of multiple beneficial PARPs would be highly desirable.

Finally, it is highly possible that any single strategy outlined above will not be good enough to fight viral infections, but a combination of all three will be effective. Thus, as progress is made on the three strategies, testing the combination of them would be very interesting. Furthermore, as we learn more about how viruses counteract the immune response mediated by NAD^+^-consuming enzymes, other therapeutic strategies may also emerge.

## Abbreviations

AAV: adeno-associated virus
AHR: aryl hydrocarbon receptor
AIV: avian influenza virus
ARTs: ADP-ribosyltransferases
BMDMs: bone-marrow derived macrophages
DTX3L: Deltex E3 ubiquitin ligase 3L
EBV: Epstein–Barr virus
EMCV: encephalomyocarditis virus
ERK: extracellular signal-regulated kinase
EXOSC5: exosome complex component 5
HA: hemagglutinin
HBV: hepatitis B virus
HSV: Herpes simplex virus
IAV: influenza A virus
ICP0: infected cell protein 0
IFNAR: interferon alpha/beta receptor
ISGs: interferon-stimulated genes
KSHV: Kaposi sarcoma-associated herpesvirus
LANA: latency-associated nuclear antigen
LPS: lipopolysaccharide
MAVS: mitochondrial antiviral-signaling
MHV: mouse hepatitis virus
MLV: murine leukemia virus
NaMN: nicotinate mononucleotide
PARPs: poly-ADP-ribose polymerases
PF: processivity factor
QPRT: quinolinate phosphoribosyltransferase
ROS: reactive oxygen species
RVFV: rift valley fever virus
SARM1: sterile alpha and TIR motif containing 1
TBK: tank binding kinase
TCDD: 2,3,7,8-tetrachlorodibenzo-p-dioxin
ZAP: zinc finger antiviral protein

## References

[1] Luengo, A., Li, Z., Gui, D.Y., Sullivan, L.B., Zagorulya, M., Do, B.T. et al. (2021) Increased demand for NAD+ relative to ATP drives aerobic glycolysis. Mol. Cell 81, 691–707.e6 10.1016/J.MOLCEL.2020.12.012 33382985 PMC8315838

[2] Corda, D. and Di Girolamo, M. (2003) Functional aspects of protein mono-ADP-ribosylation. EMBO J. 22, 1953–1958 10.1093/emboj/cdg209 12727863 PMC156081

[3] Imai, S. and Guarente, L. (2010) Ten years of NAD-dependent SIR2 family deacetylases: implications for metabolic diseases. Trends Pharmacol. Sci. 31, 212–220 10.1016/j.tips.2010.02.003 20226541 PMC3526941

[4] Du, J., Zhou, Y., Su, X., Yu, J.J., Khan, S., Jiang, H. et al. (2011) Sirt5 Is a NAD-dependent protein lysine demalonylase and desuccinylase. Science 334, 806–809 10.1126/SCIENCE.1207861 22076378 PMC3217313

[5] Imai, S., Armstrong, C.M., Kaeberlein, M. and Guarente, L. (2000) Transcriptional silencing and longevity protein Sir2 is an NAD-dependent histone deacetylase. Nature 403, 795–800 10.1038/35001622 10693811

[6] Jiang, H., Khan, S., Wang, Y., Charron, G., He, B., Sebastian, C. et al. (2013) SIRT6 regulates TNF-α secretion through hydrolysis of long-chain fatty acyl lysine. Nature 496, 110–113 10.1038/nature12038 23552949 PMC3635073

[7] Peng, C., Lu, Z., Xie, Z., Cheng, Z., Chen, Y., Tan, M. et al. (2011) The first identification of lysine malonylation substrates and its regulatory enzyme. Mol. Cell. Proteomics 10, M111.012658 10.1074/mcp.M111.012658 PMC3237090 21908771

[8] Diefenbach, J. and Bürkle, A. (2005) Poly-ADP-ribosylation in health and disease. Cell. Mol. Life Sci. 62, 721–730 10.1007/S00018-004-4503-3 15868397 PMC11924567

[9] Hassa, P.O., Haenni, S.S., Elser, M. and Hottiger, M.O. (2006) Nuclear ADP-ribosylation reactions in mammalian cells: where are we today and where are we going? Microbiol. Mol. Biol. Rev. 70, 789–829 10.1128/mmbr.00040-05 16959969 PMC1594587

[10] Groslambert, J., Prokhorova, E. and Ahel, I. (2021) ADP-ribosylation of DNA and RNA. DNA Repair (Amst) 105, 103144–103144 10.1016/J.DNAREP.2021.103144 34116477 PMC8385414

[11] Amé, J.C., Spenlehauer, C. and De Murcia, G. (2004) The PARP superfamily. Bioessays 26, 882–893 10.1002/bies.20085 15273990

[12] Vyas, S., Chesarone-Cataldo, M., Todorova, T., Huang, Y.-H.H. and Chang, P. (2013) A systematic analysis of the PARP protein family identifies new functions critical for cell physiology. Nat. Commun. 4, 2240 10.1038/ncomms3240 23917125 PMC3756671

[13] Koh, D.W., Dawson, T.M. and Dawson, V.L. (2005) Mediation of cell death by poly(ADP-ribose) polymerase-1. Pharmacol. Res. 52, 5–14 10.1016/j.phrs.2005.02.011 15911329

[14] Jwa, M. and Chang, P. (2012) PARP16 is a tail-anchored endoplasmic reticulum protein required for the PERK-and IRE1α-mediated unfolded protein response. Nat. Cell Biol. 14, 1223–1230 10.1038/ncb2593 23103912 PMC3494284

[15] Zhang, L., Cao, J., Dong, L. and Lin, H. (2020) TiPARP forms nuclear condensates to degrade HIF-1α and suppress tumorigenesis. Proc. Natl Acad. Sci. U.S.A. 117, 13447–13456 10.1073/pnas.1921815117 32482854 PMC7306777

[16] Malanga, M. and Althaus, F.R. (2005) The role of poly(ADP-ribose) in the DNA damage signaling network. Biochem. Cell Biol. 83, 354–364 10.1139/o05-038 15959561

[17] Ha, G.H., Kim, H.S., Go, H., Lee, H., Seimiya, H., Chung, D.H. et al. (2012) Tankyrase-1 function at telomeres and during mitosis is regulated by polo-like kinase-1-mediated phosphorylation. Cell Death Differ. 19, 321–332 10.1038/cdd.2011.101 21818122 PMC3263489

[18] Schreiber, V., Dantzer, F., Amé, J.C. and De Murcia, G. (2006) Poly(ADP-ribose): novel functions for an old molecule. Nat. Rev. Mol. Cell Biol. 7, 517–528 10.1038/nrm1963 16829982

[19] Hassa, P.O. and Hottiger, M.O. (2008) The diverse biological roles of mammalian PARPs, a small but powerful family of poly-ADP-ribose polymerases. Front. Biosci. 13, 3046–3082 10.2741/2909 17981777

[20] Szántó, M. and Bai, P. (2020) The role of ADP-ribose metabolism in metabolic regulation, adipose tissue differentiation, and metabolism. Genes Dev. 34, 321–340 10.1101/gad.334284.119 32029456 PMC7050491

[21] Kuny, C.V. and Sullivan, C.S. (2016) Virus–host interactions and the ARTD/PARP family of enzymes. PLoS Pathog. 12, e1005453 10.1371/journal.ppat.1005453 27010460 PMC4806868

[22] Malgras, M., Garcia, M., Jousselin, C., Bodet, C., Leveque, N. and Lévêque, N. (2021) The antiviral activities of poly-ADP-ribose polymerases. Viruses 13, 582 10.3390/v13040582 33808354 PMC8066025

[23] Funaro, A., Ortolan, E., Bovino, P., Lo Buono, N., Nacci, G., Parrotta, R. et al. (2009) Ectoenzymes and innate immunity: the role of human CD157 in leukocyte trafficking. Front. Biosci. 14, 929–943 10.2741/3287 19273109

[24] Partidá-Sánchez, S., Rivero-Nava, L., Shi, G. and Lund, F.E. (2007) CD38: an ecto-enzyme at the crossroads of innate and adaptive immune responses. Adv. Exp. Med. Biol. 590, 171–183 10.1007/978-0-387-34814-8_12 17191385

[25] Liu, Q., Graeff, R., Kriksunov, I.A., Jiang, H., Zhang, B., Oppenheimer, N. et al. (2009) Structural basis for enzymatic evolution from a dedicated ADP-ribosyl cyclase to a multifunctional NAD hydrolase. J. Biol. Chem. 284, 27637–27645 10.1074/jbc.M109.031005 19640846 PMC2785692

[26] Loring, H.S., Czech, V.L., Icso, J.D., O'connor, L., Parelkar, S., Byrne, A.B. et al. (2021) A phase transition enhances the catalytic activity of sarm1, an nad+ glycohydrolase involved in neurodegeneration. eLife 10, e66694 10.7554/eLife.66694 34184985 PMC8266388

[27] Grunewald, M.E., Shaban, M.G., Mackin, S.R., Fehr, A.R. and Perlman, S. (2020) Murine coronavirus infection activates the aryl hydrocarbon receptor in an indoleamine 2,3-dioxygenase-independent manner, contributing to cytokine modulation and proviral TCDD-inducible-PARP expression. J. Virol. 94, e01743-19 10.1128/JVI.01743-19 31694960 PMC7000979

[28] Heer, C.D., Sanderson, D.J., Voth, L.S., Alhammad, Y.M.O., Schmidt, M.S., Trammell, S.A.J. et al. (2020) Coronavirus infection and PARP expression dysregulate the NAD metabolome: An actionable component of innate immunity. J. Biol. Chem. 295, 17986–17996 10.1074/jbc.RA120.015138 33051211 PMC7834058

[29] Gerdts, J., Brace, E.J., Sasaki, Y., DiAntonio, A. and Milbrandt, J. (2015) SARM1 activation triggers axon degeneration locally via NAD^+^ destruction. Science 348, 453–457 10.1126/science.1258366 25908823 PMC4513950

[30] Panneerselvam, P., Singh, L.P., Selvarajan, V., Chng, W.J., Ng, S.B., Tan, N.S. et al. (2013) T-cell death following immune activation is mediated by mitochondria-localized SARM. Cell Death Differ. 20, 478–489 10.1038/CDD.2012.144 23175186 PMC3569988

[31] Horsefield, S., Burdett, H., Zhang, X., Manik, M.K., Shi, Y., Chen, J. et al. (2019) NAD+cleavage activity by animal and plant TIR domains in cell death pathways. Science 365, 793–799 10.1126/SCIENCE.AAX1911 31439792

[32] Wan, L., Essuman, K., Anderson, R.G., Sasaki, Y., Monteiro, F., Chung, E.H. et al. (2019) TIR domains of plant immune receptors are NAD +-cleaving enzymes that promote cell death. Science 365, 799–803 10.1126/SCIENCE.AAX1771 31439793 PMC7045805

[33] Azarm, K. and Smith, S. (2020) Nuclear PARPs and genome integrity. Genes Dev. 34, 285–301 10.1101/gad.334730.119 32029453 PMC7050482

[34] Ohsaki, E., Ueda, K., Sakakibara, S., Do, E., Yada, K. and Yamanishi, K. (2004) Poly(ADP-ribose) polymerase 1 binds to Kaposi's sarcoma-associated herpesvirus (KSHV) terminal repeat sequence and modulates KSHV replication in latency. J. Virol. 78, 9936–9946 10.1128/JVI.78.18.9936-9946.2004 15331727 PMC514965

[35] Gwack, Y., Nakamura, H., Lee, S.H., Souvlis, J., Yustein, J.T., Gygi, S. et al. (2003) Poly(ADP-ribose) polymerase 1 and Ste20-like kinase hKFC act as transcriptional repressors for gamma-2 herpesvirus lytic replication. Mol. Cell. Biol. 23, 8282–8294 10.1128/MCB.23.22.8282-8294.2003 14585985 PMC262387

[36] Romanova, L.G., Zacharias, J., Cannon, M.L. and Philpott, N.J. (2011) Effect of poly(ADP-ribose) polymerase 1 on integration of the adeno-associated viral vector genome. J. Gene Med. 13, 342–352 10.1002/JGM.1577 21674737

[37] Lupey-Green, L.N., Moquin, S.A., Martin, K.A., McDevitt, S.M., Hulse, M., Caruso, L.B. et al. (2017) PARP1 restricts Epstein Barr Virus lytic reactivation by binding the BZLF1 promoter. Virology 507, 220–230 10.1016/j.virol.2017.04.006 28456021 PMC5521201

[38] Li, Y., Wu, Y., Zheng, X., Cong, J., Liu, Y., Li, J. et al. (2016) Cytoplasm-translocated Ku70/80 complex sensing of HBV DNA induces hepatitis-associated chemokine secretion. Front. Immunol. 7, 569 10.3389/fimmu.2016.00569 27994596 PMC5136554

[39] Chung, W.C., Park, J.H., Kang, H.R. and Song, M.J. (2015) Downregulation of poly(ADP-ribose) polymerase 1 by a viral processivity factor facilitates lytic replication of gammaherpesvirus. J. Virol. 89, 9676–9682 10.1128/JVI.00559-15 26157130 PMC4542354

[40] Noh, C.W., Cho, H.J., Kang, H.R., Jin, H.Y., Lee, S., Deng, H. et al. (2012) The virion-associated open reading frame 49 of murine gammaherpesvirus 68 promotes viral replication both in vitro and in vivo as a derepressor of RTA. J. Virol. 86, 1109–1118 10.1128/JVI.05785-11 22090108 PMC3255801

[41] Yu, D., Liu, R., Yang, G. and Zhou, Q. (2018) The PARP1-Siah1 axis controls HIV-1 transcription and expression of Siah1 substrates. Cell Rep. 23, 3741–3749 10.1016/j.celrep.2018.05.084 29949759 PMC6223328

[42] Ha, H.C., Juluri, K., Zhou, Y., Leung, S., Hermankova, M. and Snyder, S.H. (2001) Poly(ADP-ribose) polymerase-1 is required for efficient HIV-1 integration. Proc. Natl Acad. Sci. U.S.A. 98, 3364–3368 10.1073/pnas.051633498 11248084 PMC30659

[43] Kameoka, M., Nukuzuma, S., Itaya, A., Tanaka, Y., Ota, K., Ikuta, K. et al. (2004) RNA interference directed against poly(ADP-Ribose) polymerase 1 efficiently suppresses human immunodeficiency virus type 1 replication in human cells. J. Virol. 78, 8931–8934 10.1128/JVI.78.16.8931-8934.2004 15280503 PMC479071

[44] Kameoka, M., Nukuzuma, S., Itaya, A., Tanaka, Y., Ota, K., Inada, Y. et al. (2005) Poly(ADP-ribose)polymerase-1 is required for integration of the human immunodeficiency virus type 1 genome near centromeric alphoid DNA in human and murine cells. Biochem. Biophys. Res. Commun. 334, 412–417 10.1016/j.bbrc.2005.06.104 16002043

[45] Rom, S., Reichenbach, N.L., Dykstra, H. and Persidsky, Y. (2015) The dual action of poly(ADP-ribose) polymerase -1 (PARP-1) inhibition in HIV-1 infection: HIV-1 LTR inhibition and diminution in Rho GTPase activity. Front. Microbiol. 6, 878 10.3389/fmicb.2015.00878 26379653 PMC4548080

[46] Bueno, M.T., Reyes, D., Valdes, L., Saheba, A., Urias, E., Mendoza, C. et al. (2013) Poly(ADP-ribose) polymerase 1 promotes transcriptional repression of integrated retroviruses. J. Virol. 87, 2496–2507 10.1128/JVI.01668-12 23255787 PMC3571415

[47] Gutierrez, D.A., Valdes, L., Serguera, C. and Llano, M. (2016) Poly(ADP-ribose) polymerase-1 silences retroviruses independently of viral DNA integration or heterochromatin formation. J. Gen. Virol. 97, 1686–1692 10.1099/jgv.0.000466 27028089 PMC7011754

[48] Ariumi, Y., Turelli, P., Masutani, M. and Trono, D. (2005) DNA damage sensors ATM, ATR, DNA-PKcs, and PARP-1 are dispensable for human immunodeficiency virus type 1 integration. J. Virol. 79, 2973–2978 10.1128/JVI.79.5.2973-2978.2005 15709017 PMC548471

[49] Siva, A.C. and Bushman, F. (2002) Poly(ADP-ribose) polymerase 1 is not strictly required for infection of murine cells by retroviruses. J. Virol. 76, 11904–11910 10.1128/jvi.76.23.11904-11910.2002 12414932 PMC136881

[50] Xia, C., Wolf, J.J., Sun, C., Xu, M., Studstill, C.J., Chen, J. et al. (2020) PARP1 enhances influenza A virus propagation by facilitating degradation of host type I interferon receptor. J. Virol. 94, e01572-19 10.1128/JVI.01572-19 PMC7081902 31915279

[51] Bortz, E., Westera, L., Maamary, J., Steel, J., Albrecht, R.A., Manicassamy, B. et al. (2011) Host- and strain-specific regulation of influenza virus polymerase activity by interacting cellular proteins. MBio 2, e00151-11 10.1128/mBio.00151-11 21846828 PMC3157893

[52] Parent, M., Yung, T.M., Rancourt, A., Ho, E.L., Vispe, S., Suzuki-Matsuda, F. et al. (2005) Poly(ADP-ribose) polymerase-1 is a negative regulator of HIV-1 transcription through competitive binding to TAR RNA with Tat.positive transcription elongation factor b (p-TEFb) complex. J. Biol. Chem. 280, 448–457 10.1074/jbc.M408435200 15498776

[53] Li, Z., Yamauchi, Y., Kamakura, M., Murayama, T., Goshima, F., Kimura, H. et al. (2012) Herpes simplex virus requires poly(ADP-ribose) polymerase activity for efficient replication and induces extracellular signal-related kinase-dependent phosphorylation and ICP0-dependent nuclear localization of tankyrase 1. J. Virol. 86, 492–503 10.1128/JVI.05897-11 22013039 PMC3255871

[54] Deng, Z., Lezina, L., Chen, C.-J., Shtivelband, S., So, W. and Lieberman, P.M. (2002) Telomeric proteins regulate episomal maintenance of Epstein-Barr virus origin of plasmid replication. Mol. Cell 9, 493–503 10.1016/s1097-2765(02)00476-8 11931758

[55] Deng, Z., Atanasiu, C., Zhao, K., Marmorstein, R., Sbodio, J.I., Chi, N.W. et al. (2005) Inhibition of Epstein-Barr virus OriP function by tankyrase, a telomere-associated poly-ADP ribose polymerase that binds and modifies EBNA1. J. Virol. 79, 4640–4650 10.1128/JVI.79.8.4640-4650.2005 15795250 PMC1069541

[56] Smith, S., Giriat, I., Schmitt, A. and de Lange, T. (1998) Tankyrase, a poly(ADP-ribose) polymerase at human telomeres. Science 282, 1484 10.1126/science.282.5393.1484 9822378

[57] Tempera, I., Deng, Z., Atanasiu, C., Chen, C.J., D'Erme, M. and Lieberman, P.M. (2010) Regulation of Epstein-Barr virus OriP replication by poly(ADP-ribose) polymerase 1. J. Virol. 84, 4988–4997 10.1128/JVI.02333-09 20219917 PMC2863838

[58] Ma, Q. (2002) Induction and superinduction of 2,3,7,8-tetrachlorodibenzo-p-dioxin-inducible poly(ADP-ribose) polymerase: Role of the aryl hydrocarbon receptor/aryl hydrocarbon receptor nuclear translocator transcription activation domains and a labile transcription rep. Arch. Biochem. Biophys. 404, 309–316 10.1016/S0003-9861(02)00339-9 12147270

[59] Ma, Q., Baldwin, K.T., Renzelli, A.J., McDaniel, A. and Dong, L. (2001) TCDD-inducible poly(ADP-ribose) polymerase: A novel response to 2,3,7,8-Tetrachlorodibenzo-p-dioxin. Biochem. Biophys. Res. Commun. 289, 499–506 10.1006/bbrc.2001.5987 11716501

[60] Atasheva, S., Frolova, E.I. and Frolov, I. (2014) Interferon-stimulated poly(ADP-Ribose) polymerases are potent inhibitors of cellular translation and virus replication. J. Virol. 88, 2116–2130 10.1128/JVI.03443-13 24335297 PMC3911523

[61] Kozaki, T., Komano, J., Kanbayashi, D., Takahama, M., Misawa, T., Satoh, T. et al. (2017) Mitochondrial damage elicits a TCDD-inducible poly(ADP-ribose) polymerase-mediated antiviral response. Proc. Natl Acad. Sci. U.S.A. 114, 2681–2686 10.1073/pnas.1621508114 28213497 PMC5347618

[62] Grunewald, M.E., Chen, Y., Kuny, C., Maejima, T., Lease, R., Ferraris, D. et al. (2019) The coronavirus macrodomain is required to prevent PARP-mediated inhibition of virus replication and enhancement of IFN expression. PLoS Pathog. 15, e1007756 10.1371/journal.ppat.1007756 31095648 PMC6521996

[63] Bolton, E.C., So, A.Y., Chaivorapol, C., Haqq, C.M., Li, H. and Yamamoto, K.R. (2007) Cell- and gene-specific regulation of primary target genes by the androgen receptor. Genes Dev. 21, 2005–2017 10.1101/gad.1564207 17699749 PMC1948856

[64] Rasmussen, M., Tan, S., Somisetty, V.S., Hutin, D., Olafsen, N.E., Moen, A. et al. (2021) PARP7 and mono-ADP-ribosylation negatively regulate estrogen receptor α signaling in human breast cancer cells. Cells 10, 623 10.3390/cells10030623 33799807 PMC8001409

[65] Kamata, T., Yang, C.S., Melhuish, T.A., Frierson, H.F., Wotton, D. and Paschal, B.M. (2021) Post-transcriptional regulation of PARP7 protein stability Is controlled by androgen signaling. Cells 10, 363 10.3390/cells10020363 33572475 PMC7916378

[66] Rodriguez, K.M., Buch-Larsen, S.C., Kirby, I.T., Siordia, I.R., Hutin, D., Rasmussen, M. et al. (2021) Chemical genetics and proteome-wide site mapping reveal cysteine MARylation by PARP-7 on immune-relevant protein targets. eLife 10, e60480 10.7554/eLife.60480 33475084 PMC7880690

[67] Yamada, T., Horimoto, H., Kameyama, T., Hayakawa, S., Yamato, H., Dazai, M. et al. (2016) Constitutive aryl hydrocarbon receptor signaling constrains type I interferon-mediated antiviral innate defense. Nat. Immunol. 17, 687–694 10.1038/ni.3422 27089381

[68] Rijo, M.P., Diani-Moore, S., Yang, C., Zhou, P. and Rifkind, A.B. (2021) Roles of the ubiquitin ligase CUL4B and ADP-ribosyltransferase TiPARP in TCDD-induced nuclear export and proteasomal degradation of the transcription factor AHR. J. Biol. Chem. 297, 100886 10.1016/j.jbc.2021.100886 34146543 PMC8318916

[69] Liang, J., Song, W., Tromp, G., Kolattukudy, P.E. and Fu, M. (2008) Genome-wide survey and expression profiling of CCCH-zinc finger family reveals a functional module in macrophage activation. PLoS ONE 3, e2880 10.1371/journal.pone.0002880 18682727 PMC2478707

[70] Zhang, Y., Mao, D., Roswit, W.T., Jin, X., Patel, A.C., Patel, D.A. et al. (2015) PARP9-DTX3L ubiquitin ligase targets host histone H2BJ and viral 3C protease to enhance interferon signaling and control viral infection. Nat. Immunol. 16, 1215–1227 10.1038/ni.3279 26479788 PMC4653074

[71] Xing, J., Zhang, A., Du, Y., Fang, M., Minze, L.J., Liu, Y.J. et al. (2021) Identification of poly(ADP-ribose) polymerase 9 (PARP9) as a noncanonical sensor for RNA virus in dendritic cells. Nat. Commun. 12, 2681 10.1038/s41467-021-23003-4 33976210 PMC8113569

[72] Yu, M., Zhang, C., Yang, Y., Yang, Z., Zhao, L., Xu, L. et al. (2011) The interaction between the PARP10 protein and the NS1 protein of H5N1 AIV and its effect on virus replication. Virol. J. 8, 546 10.1186/1743-422X-8-546 22176891 PMC3287249

[73] Guo, T., Zuo, Y., Qian, L., Liu, J., Yuan, Y., Xu, K. et al. (2019) ADP-ribosyltransferase PARP11 modulates the interferon antiviral response by mono-ADP-ribosylating the ubiquitin E3 ligase beta-TrCP. Nat. Microbiol. 4, 1872–1884 10.1038/s41564-019-0428-3 30988430

[74] Li, L., Shi, Y., Li, S., Liu, J., Zu, S., Xu, X. et al. (2021) ADP-ribosyltransferase PARP11 suppresses Zika virus in synergy with PARP12. Cell Biosci. 11, 116 10.1186/s13578-021-00628-y 34187568 PMC8240438

[75] Guo, X., Carroll, J.-W.N., MacDonald, M.R., Goff, S.P. and Gao, G. (2004) The zinc finger antiviral protein directly binds to specific viral mRNAs through the CCCH zinc finger motifs. J. Virol. 78, 12781–12787 10.1128/jvi.78.23.12781-12787.2004 15542630 PMC525010

[76] Guo, X., Ma, J., Sun, J. and Gao, G. (2007) The zinc-finger antiviral protein recruits the RNA processing exosome to degrade the target mRNA. Proc. Natl Acad. Sci. U.S.A. 104, 151–156 10.1073/pnas.0607063104 17185417 PMC1765426

[77] Hall, T.M.T. (2005) Multiple modes of RNA recognition by zinc finger proteins. Curr. Opin. Struct. Biol. 15, 367–373 10.1016/j.sbi.2005.04.004 15963892

[78] Liu, S.Y., Sanchez, D.J., Aliyari, R., Lu, S. and Cheng, G. (2012) Systematic identification of type I and type II interferon-induced antiviral factors. Proc. Natl Acad. Sci. U.S.A. 109, 4239–4244 10.1073/pnas.1114981109 22371602 PMC3306696

[79] Miettinen, M., Vedantham, M. and Pulliainen, A.T. (2019) Host poly(ADP-ribose) polymerases (PARPs) in acute and chronic bacterial infections. Microbes Infect. 21, 423–431 10.1016/j.micinf.2019.06.002 31207286

[80] Atasheva, S., Akhrymuk, M., Frolova, E.I. and Frolov, I. (2012) New PARP gene with an anti-alphavirus function. J. Virol. 86, 8147–8160 10.1128/jvi.00733-12 22623789 PMC3421642

[81] Li, L., Zhao, H., Liu, P., Li, C., Quanquin, N., Ji, X. et al. (2018) PARP12 suppresses Zika virus infection through PARP-dependent degradation of NS1 and NS3 viral proteins. Sci. Signal. 11, eaas9332 10.1126/scisignal.aas9332 29921658 PMC6434931

[82] Welsby, I., Hutin, D., Gueydan, C., Kruys, V., Rongvaux, A. and Leo, O. (2014) PARP12, an interferon-stimulated gene involved in the control of protein translation and inflammation. J. Biol. Chem. 289, 26642–26657 10.1074/jbc.M114.589515 25086041 PMC4176246

[83] Lee, S., Lee, Y.S., Choi, Y., Son, A., Park, Y., Lee, K.M. et al. (2021) The SARS-CoV-2 RNA interactome. Mol. Cell 81, 2838–2850.e6 10.1016/J.MOLCEL.2021.04.022 33989516 PMC8075806

[84] Catara, G., Grimaldi, G., Schembri, L., Spano, D., Turacchio, G., Lo Monte, M. et al. (2017) PARP1-produced poly-ADP-ribose causes the PARP12 translocation to stress granules and impairment of Golgi complex functions. Sci. Rep. 7, 14035 10.1038/s41598-017-14156-8 29070863 PMC5656619

[85] Miyazato, P., Matsuo, M., Tan, B.J.Y., Tokunaga, M., Katsuya, H., Islam, S. et al. (2019) HTLV-1 contains a high CG dinucleotide content and is susceptible to the host antiviral protein ZAP. Retrovirology 16, 38 10.1186/s12977-019-0500-3 31842935 PMC6915898

[86] Chiu, H.P., Chiu, H., Yang, C.F., Lee, Y.L., Chiu, F.L., Kuo, H.C. et al. (2018) Inhibition of Japanese encephalitis virus infection by the host zinc-finger antiviral protein. PLoS Pathog. 14, e1007166 10.1371/journal.ppat.1007166 30016363 PMC6049953

[87] Zhao, Y., Song, Z., Bai, J., Liu, X., Nauwynck, H. and Jiang, P. (2019) ZAP, a CCCH-type zinc finger protein, inhibits porcine reproductive and respiratory syndrome virus replication and interacts with viral Nsp9. J. Virol. 93, e00001-19 10.1128/jvi.00001-19 30867303 PMC6498049

[88] Liu, C.H., Zhou, L., Chen, G. and Krug, R.M. (2015) Battle between influenza A virus and a newly identified antiviral activity of the PARP-containing ZAPL protein. Proc. Natl Acad. Sci. U.S.A. 112, 14048–14053 10.1073/pnas.1509745112 26504237 PMC4653199

[89] Mao, R., Nie, H., Cai, D., Zhang, J., Liu, H., Yan, R. et al. (2013) Inhibition of hepatitis B virus replication by the host zinc finger antiviral protein. PLoS Pathog. 9, e1003494 10.1371/journal.ppat.1003494 23853601 PMC3708887

[90] Zhu, Y., Chen, G., Lv, F., Wang, X., Ji, X., Xu, Y. et al. (2011) Zinc-finger antiviral protein inhibits HIV-1 infection by selectively targeting multiply spliced viral mRNAs for degradation. Proc. Natl Acad. Sci. U.S.A. 108, 15834–15839 10.1073/pnas.1101676108 21876179 PMC3179061

[91] Xuan, Y., Gong, D., Qi, J., Han, C., Deng, H. and Gao, G. (2013) ZAP inhibits murine gammaherpesvirus 68 ORF64 expression and is antagonized by RTA. J. Virol. 87, 2735–2743 10.1128/jvi.03015-12 23255809 PMC3571413

[92] Bick, M.J., Carroll, J.W., Gao, G., Goff, S.P., Rice, C.M. and MacDonald, M.R. (2003) Expression of the zinc-finger antiviral protein inhibits alphavirus replication. J. Virol. 77, 11555–11562 10.1128/jvi.77.21.11555-11562.2003 14557641 PMC229374

[93] Kerns, J.A., Emerman, M. and Malik, H.S. (2008) Positive selection and increased antiviral activity associated with the PARP-containing isoform of human zinc-finger antiviral protein. PLoS Genet. 4, e21 10.1371/journal.pgen.0040021 18225958 PMC2213710

[94] Goodier, J.L., Pereira, G.C., Cheung, L.E., Rose, R.J., Kazazian, H.H. and Kazazian, Jr, H.H. (2015) The broad-spectrum antiviral protein ZAP restricts human retrotransposition. PLoS Genet. 11, e1005252 10.1371/journal.pgen.1005252 26001115 PMC4441479

[95] Su, C., Zhang, J. and Zheng, C. (2015) Herpes simplex virus 1 UL41 protein abrogates the antiviral activity of hZAP by degrading its mRNA. Virol. J. 12, 203 10.1186/s12985-015-0433-y 26625984 PMC4666169

[96] Xie, L., Lu, B., Zheng, Z., Miao, Y., Liu, Y., Zhang, Y. et al. (2018) The 3C protease of enterovirus A71 counteracts the activity of host zinc-finger antiviral protein (ZAP). J. Gen. Virol. 99, 73–85 10.1099/jgv.0.000982 29182509

[97] Ficarelli, M., Neil, S.J.D. and Swanson, C.M. (2021) Targeted restriction of viral gene expression and replication by the ZAP antiviral system. Annu. Rev. Virol. 8, 265–283 10.1146/annurev-virology-091919-104213 34129371

[98] Hayakawa, S., Shiratori, S., Yamato, H., Kameyama, T., Kitatsuji, C., Kashigi, F. et al. (2011) ZAPS is a potent stimulator of signaling mediated by the RNA helicase RIG-I during antiviral responses. Nat. Immunol. 12, 37–44 10.1038/ni.1963 21102435

[99] Gao, G., Guo, X. and Goff, S.P. (2002) Inhibition of retroviral RNA production by ZAP, a CCCH-type zinc finger protein. Science 297, 1703–1706 10.1126/science.1074276 12215647

[100] Takata, M.A., Gonçalves-Carneiro, D., Zang, T.M., Soll, S.J., York, A., Blanco-Melo, D. et al. (2017) CG dinucleotide suppression enables antiviral defence targeting non-self RNA. Nature 550, 124–127 10.1038/nature24039 28953888 PMC6592701

[101] Zhu, Y., Wang, X., Goff, S.P. and Gao, G. (2012) Translational repression precedes and is required for ZAP-mediated mRNA decay. EMBO J. 31, 4236–4246 10.1038/emboj.2012.271 23023399 PMC3492732

[102] Law, L.M.J., Razooky, B.S., Li, M.M.H., You, S., Jurado, A., Rice, C.M. et al. (2019) ZAP's stress granule localization is correlated with its antiviral activity and induced by virus replication. PLoS Pathog. 15, e1007798 10.1371/journal.ppat.1007798 31116799 PMC6548403

[103] Nchioua, R., Kmiec, D., Müller, J.A., Conzelmann, C., Groß, R., Swanson, C.M. et al. (2020) Sars-cov-2 is restricted by zinc finger antiviral protein despite preadaptation to the low-cpg environment in humans. MBio 11, e01930-20 10.1128/mBio.01930-20 33067384 PMC7569149

[104] Sadykov, M., Mourier, T., Guan, Q. and Pain, A. (2020) Short sequence motif dynamics in the SARS-CoV-2 genome suggest a role for cytosine deamination in CpG reduction. J. Mol. Cell Biol. 13, 225–227 10.1101/2020.06.19.161687 PMC7928816 33630074

[105] Azgari, C., Kilinc, Z., Turhan, B., Circi, D. and Adebali, O. (2021) The mutation profile of sars-cov-2 is primarily shaped by the host antiviral defense. Viruses 13, 394 10.3390/v13030394 33801257 PMC7999997

[106] Todorova, T., Bock, F.J. and Chang, P. (2014) PARP13 regulates cellular mRNA post-transcriptionally and functions as a pro-apoptotic factor by destabilizing TRAILR4 transcript. Nat. Commun. 5, 5362 10.1038/ncomms6362 25382312 PMC4228382

[107] Seo, G.J., Kincaid, R.P., Phanaksri, T., Burke, J.M., Pare, J.M., Cox, J.E. et al. (2013) Reciprocal inhibition between intracellular antiviral signaling and the RNAi machinery in mammalian cells. Cell Host Microbe 14, 435–445 10.1016/j.chom.2013.09.002 24075860 PMC3837626

[108] Iwata, H., Goettsch, C., Sharma, A., Ricchiuto, P., Goh, W.W., Halu, A. et al. (2016) PARP9 and PARP14 cross-regulate macrophage activation via STAT1 ADP-ribosylation. Nat. Commun. 7, 12849 10.1038/ncomms12849 27796300 PMC5095532

[109] Caprara, G., Prosperini, E., Piccolo, V., Sigismondo, G., Melacarne, A., Cuomo, A. et al. (2018) PARP14 controls the nuclear accumulation of a subset of type I IFN-inducible proteins. J. Immunol. 200, 2439–2454 10.4049/jimmunol.1701117 29500242

[110] Fehr, A.R., Jankevicius, G., Ahel, I. and Perlman, S. (2018) Viral macrodomains: unique mediators of viral replication and pathogenesis. Trends Microbiol. 26, 598–610 10.1016/j.tim.2017.11.011 29268982 PMC6003825

[111] Eckei, L., Krieg, S., Butepage, M., Lehmann, A., Gross, A., Lippok, B. et al. (2017) The conserved macrodomains of the non-structural proteins of Chikungunya virus and other pathogenic positive strand RNA viruses function as mono-ADP-ribosylhydrolases. Sci. Rep. 7, 41746 10.1038/srep41746 28150709 PMC5288732

[112] Fehr, A.R., Channappanavar, R., Jankevicius, G., Fett, C., Zhao, J., Athmer, J. et al. (2016) The conserved coronavirus macrodomain promotes virulence and suppresses the innate immune response during severe acute respiratory syndrome coronavirus infection. MBio 7, e01721-16 10.1128/mBio.01721-16 27965448 PMC5156301

[113] Li, C., Debing, Y., Jankevicius, G., Neyts, J., Ahel, I., Coutard, B. et al. (2016) Viral macro domains reverse protein ADP-ribosylation. J. Virol. 90, 8478–8486 10.1128/JVI.00705-16 27440879 PMC5021415

[114] Kuri, T., Eriksson, K.K., Putics, A., Zust, R., Snijder, E.J., Davidson, A.D. et al. (2011) The ADP-ribose-1″-monophosphatase domains of severe acute respiratory syndrome coronavirus and human coronavirus 229E mediate resistance to antiviral interferon responses. J. Gen. Virol. 92, 1899–1905 10.1099/vir.0.031856-0 21525212

[115] Webb, T.E. and Saad, R. (2020) Sequence homology between human PARP14 and the SARS-CoV-2 ADP ribose 1’-phosphatase. Immunol. Lett. 224, 38–39 10.1016/j.imlet.2020.05.008 32534867 PMC7289111

[116] Haigis, M.C. and Sinclair, D.A. (2010) Mammalian sirtuins: biological insights and disease relevance. Annu. Rev. Pathol. 5, 253–295 10.1146/ANNUREV.PATHOL.4.110807.092250 20078221 PMC2866163

[117] Wang, M. and Lin, H. (2021) Understanding the function of mammalian sirtuins and protein lysine acylation. Annu. Rev. Biochem. 90, 245–285 10.1146/ANNUREV-BIOCHEM-082520-125411 33848425

[118] Koyuncu, E., Budayeva, H.G., Miteva, Y.V., Ricci, D.P., Silhavy, T.J., Shenk, T. et al. (2014) Sirtuins are evolutionarily conserved viral restriction factors. MBio 5, e02249-14 10.1128/mBio.02249-14 25516616 PMC4271551

[119] Lim, H.W., Kang, S.G., Ryu, J.K., Schilling, B., Fei, M., Lee, I.S. et al. (2015) SIRT1 deacetylates RORγt and enhances Th17 cell generation. J. Exp. Med. 212, 607–617 10.1084/jem.20132378 25918343 PMC4419343

[120] Das, D., Smith, N., Wang, X. and Morgan, I.M. (2017) The deacetylase SIRT1 regulates the replication properties of human papillomavirus 16 E1 and E2. J. Virol. 91, e00102-17 10.1128/jvi.00102-17 28275188 PMC5411580

[121] Han, Y., Wang, L., Cui, J., Song, Y., Luo, Z., Chen, J. et al. (2016) SIRT1 inhibits EV71 genome replication and RNA translation by interfering with the viral polymerase and 5'UTR RNA. J. Cell Sci. 129, 4534–4547 10.1242/jcs.193698 27875274 PMC5201017

[122] Van Gool, F., Gallí, M., Gueydan, C., Kruys, V., Prevot, P.P., Bedalov, A. et al. (2009) Intracellular NAD levels regulate tumor necrosis factor protein synthesis in a sirtuin-dependent manner. Nat. Med. 15, 206–210 10.1038/nm.1906 19151729 PMC2845476

[123] Lasigliè, D., Boero, S., Bauer, I., Morando, S., Damonte, P., Cea, M. et al. (2016) Sirt6 regulates dendritic cell differentiation, maturation, and function. Aging (Albany NY) 8, 34–49 10.18632/aging.100870 26761436 PMC4761712

[124] Dantoft, W., Robertson, K.A., Watkins, W.J., Strobl, B. and Ghazal, P. (2019) Metabolic regulators Nampt and Sirt6 serially participate in the macrophage interferon antiviral cascade. Front. Microbiol. 10, 355 10.3389/fmicb.2019.00355 30886604 PMC6409323

[125] Latifkar, A., Ling, L., Hingorani, A., Johansen, E., Clement, A., Zhang, X. et al. (2019) Loss of sirtuin 1 alters the secretome of breast cancer cells by impairing lysosomal integrity. Dev. Cell 49, 393–408.e7 10.1016/j.devcel.2019.03.011 30982660 PMC6519475

[126] Salminen, A. and Kaarniranta, K. (2009) SIRT1: regulation of longevity via autophagy. Cell Signal. 21, 1356–1360 10.1016/j.cellsig.2009.02.014 19249351

[127] Mao, J., Lin, E., He, L., Yu, J., Tan, P. and Zhou, Y. (2019) Autophagy and viral infection. Adv. Exp. Med. Biol. 1209, 55–78 10.1007/978-981-15-0606-2_5 31728865 PMC7122562

[128] Someya, S., Yu, W., Hallows, W.C., Xu, J., Vann, J.M., Leeuwenburgh, C. et al. (2010) Sirt3 mediates reduction of oxidative damage and prevention of age-related hearing loss under caloric restriction. Cell 143, 802–812 10.1016/j.cell.2010.10.002 21094524 PMC3018849

[129] Zhou, L., Wang, F., Sun, R., Chen, X., Zhang, M., Xu, Q. et al. (2016) SIRT5 promotes IDH2 desuccinylation and G6PD deglutarylation to enhance cellular antioxidant defense. EMBO Rep. 17, 811–822 10.15252/embr.201541643 27113762 PMC5278614

[130] Lambeth, J.D. (2004) NOX enzymes and the biology of reactive oxygen. Nat. Rev. Immunol. 4, 181–189 10.1038/nri1312 15039755

[131] Glaría, E. and Valledor, A.F. (2020) Roles of CD38 in the immune response to infection. Cells 9, 228 10.3390/cells9010228 PMC7017097 31963337

[132] Rodríguez-Alba, J.C., Abrego-Peredo, A., Gallardo-Hernández, C., Pérez-Lara, J., Santiago-Cruz, W., Jiang, W. et al. (2019) HIV disease progression: overexpression of the ectoenzyme CD38 as a contributory factor? Bioessays 41, e1800128 10.1002/bies.201800128 30537007 PMC6545924

[133] Zidovec Lepej, S., Vince, A., Dakovic Rode, O., Remenar, A. and Jeren, T. (2003) Increased numbers of CD38 molecules on bright CD8+ T lymphocytes in infectious mononucleosis caused by Epstein-Barr virus infection. Clin. Exp. Immunol. 133, 384–390 10.1046/j.1365-2249.2003.02219.x 12930365 PMC1808801

[134] Malavasi, F., Deaglio, S., Funaro, A., Ferrero, E., Horenstein, A.L., Ortolan, E. et al. (2008) Evolution and function of the ADP ribosyl cyclase/CD38 gene family in physiology and pathology. Physiol. Rev. 88, 841–886 10.1152/physrev.00035.2007 18626062

[135] Lischke, T., Heesch, K., Schumacher, V., Schneider, M., Haag, F., Koch-Nolte, F. et al. (2013) CD38 controls the innate immune response against Listeria monocytogenes. Infect. Immun. 81, 4091–4099 10.1128/iai.00340-13 23980105 PMC3811837

[136] Partida-Sánchez, S., Cockayne, D.A., Monard, S., Jacobson, E.L., Oppenheimer, N., Garvy, B. et al. (2001) Cyclic ADP-ribose production by CD38 regulates intracellular calcium release, extracellular calcium influx and chemotaxis in neutrophils and is required for bacterial clearance in vivo. Nat. Med. 7, 1209–1216 10.1038/nm1101-1209 11689885

[137] Viegas, M.S., do Carmo, A., Silva, T., Seco, F., Serra, V., Lacerda, M. et al. (2007) CD38 plays a role in effective containment of mycobacteria within granulomata and polarization of Th1 immune responses against *Mycobacterium avium*. Microbes Infect. 9, 847–854 10.1016/j.micinf.2007.03.003 17533152

[138] Yakymiv, Y., Augeri, S., Fissolo, G., Peola, S., Bracci, C., Binaschi, M. et al. (2019) CD157: from myeloid cell differentiation marker to therapeutic target in acute myeloid leukemia. Cells 8, 1580 10.3390/cells8121580 PMC6952987 31817547

[139] Carty, M., Goodbody, R., Schröder, M., Stack, J., Moynagh, P.N. and Bowie, A.G. (2006) The human adaptor SARM negatively regulates adaptor protein TRIF-dependent toll-like receptor signaling. Nat. Immunol. 7, 1074–1081 10.1038/ni1382 16964262

[140] Peng, J., Yuan, Q., Lin, B., Panneerselvam, P., Wang, X., Luan, X.L. et al. (2010) SARM inhibits both TRIF- and MyD88-mediated AP-1 activation. Eur. J. Immunol. 40, 1738–1747 10.1002/EJI.200940034 20306472

[141] Krauss, R., Bosanac, T., Devraj, R., Engber, T. and Hughes, R.O. (2020) Axons matter: the promise of treating neurodegenerative disorders by targeting SARM1-mediated axonal degeneration. Trends Pharmacol. Sci. 41, 281–293 10.1016/J.TIPS.2020.01.006 32107050

[142] Sundaramoorthy, V., Green, D., Locke, K., O'Brien, C.M., Dearnley, M. and Bingham, J. (2020) Novel role of SARM1 mediated axonal degeneration in the pathogenesis of rabies. PLoS Pathog. 16, e1008343 10.1371/JOURNAL.PPAT.1008343 32069324 PMC7048299

[143] Hopkins, E.L., Gu, W., Kobe, B. and Coleman, M.P. (2021) A novel NAD signaling mechanism in axon degeneration and its relationship to innate immunity. Front. Mol. Biosci. 8, 703532 10.3389/FMOLB.2021.703532 34307460 PMC8295901

[144] Carty, M., Kearney, J., Shanahan, K.A., Hams, E., Sugisawa, R., Connolly, D. et al. (2019) Cell survival and cytokine release after inflammasome activation is regulated by the Toll-IL-1R protein SARM. Immunity 50, 1412–1424.e6 10.1016/J.IMMUNI.2019.04.005 31076360

[145] Rongvaux, A., Andris, F., Van Gool, F. and Leo, O. (2003) Reconstructing eukaryotic NAD metabolism. Bioessays 25, 683–690 10.1002/bies.10297 12815723

[146] Bieganowski, P. and Brenner, C. (2004) Discoveries of nicotinamide riboside as a nutrient and conserved NRK genes establish a Preiss-Handler independent route to NAD+ in fungi and humans. Cell 117, 495–502 10.1016/s0092-8674(04)00416-7 15137942

[147] Moni, M.A. and Lio, P. (2017) Genetic profiling and comorbidities of Zika infection. J. Infect. Dis. 216, 703–712 10.1093/infdis/jix327 28934431

[148] Bergh, V.D., Florence, R., Vlieghe, E., Boonefaes, E., Grooten, T., Houthuys, J., (2010) Transcriptome analysis of monocyte-HIV interactions. Retrovirology 7, 53 10.1186/1742-4690-7-53 20546557 PMC2900222

[149] Ramirez, R.N., El-Ali, N.C., Mager, M.A., Wyman, D., Conesa, A. and Mortazavi, A. (2017) Dynamic gene regulatory networks of human myeloid differentiation. Cell Syst. 4, 416–429.e3 10.1016/j.cels.2017.03.005 28365152 PMC5490374

[150] van der Putten, C., Veth, J., Sukurova, L., Zuiderwijk-Sick, E.A., Simonetti, E., Koenen, H., (2019) TLR-Induced IL-12 and CCL2 production by myeloid cells is dependent on adenosine A(3) receptor-mediated signaling. J. Immunol. 202, 2421–2430 10.4049/jimmunol.1800618 30804043

[151] Yu, W., Ramakrishnan, R., Wang, Y., Chiang, K., Sung, T.L. and Rice, A.P. (2008) Cyclin T1-dependent genes in activated CD4T and macrophage cell lines appear enriched in HIV-1 co-factors. PLoS ONE 3, e3146 10.1371/journal.pone.0003146 18773076 PMC2519787

[152] Chemin, K., Ramsköld, D., Diaz-Gallo, L.M., Herrath, J., Houtman, M., Tandre, K. et al. (2018) EOMES-positive CD4(+) T cells are increased in PTPN22 (1858T) risk allele carriers. Eur. J. Immunol. 48, 655–669 10.1002/eji.201747296 29388193

[153] Drewes, J.L., Croteau, J.D., Shirk, E.N., Engle, E.L., Zink, M.C. and Graham, D.R. (2016) Distinct patterns of tryptophan maintenance in tissues during kynurenine pathway activation in simian immunodeficiency virus-infected macaques. Front. Immunol. 7, 605 10.3389/fimmu.2016.00605 28066416 PMC5165277

[154] Raniga, K. and Liang, C. (2018) Interferons: reprogramming the metabolic network against viral infection. Viruses 10, 36 10.3390/v10010036 PMC5795449 29342871

[155] Bekkering, S., Arts, R.J.W., Novakovic, B., Kourtzelis, I., van der Heijden, C., Li, Y. et al. (2018) Metabolic induction of trained immunity through the mevalonate pathway. Cell 172, 135–146.e9 10.1016/j.cell.2017.11.025 29328908

[156] Galamb, O., Sipos, F., Solymosi, N., Spisák, S., Krenács, T., Tóth, K. et al. (2008) Diagnostic mRNA expression patterns of inflamed, benign, and malignant colorectal biopsy specimen and their correlation with peripheral blood results. Cancer Epidemiol. Biomarkers Prev. 17, 2835–2845 10.1158/1055-9965.epi-08-0231 18843029

[157] Lewis, N.D., Patnaude, L.A., Pelletier, J., Souza, D.J., Lukas, S.M., King, F.J. et al. (2014) A GPBAR1 (TGR5) small molecule agonist shows specific inhibitory effects on myeloid cell activation in vitro and reduces experimental autoimmune encephalitis (EAE) in vivo. PLoS ONE 9, e100883 10.1371/journal.pone.0100883 24967665 PMC4072711

[158] Pai, A.A., Baharian, G., Pagé Sabourin, A., Brinkworth, J.F., Nédélec, Y., Foley, J.W. et al. (2016) Widespread shortening of 3’ untranslated regions and increased exon inclusion are evolutionarily conserved features of innate immune responses to infection. PLoS Genet. 12, e1006338 10.1371/journal.pgen.1006338 27690314 PMC5045211

[159] Smith, A.M., Rahman, F.Z., Hayee, B., Graham, S.J., Marks, D.J., Sewell, G.W. et al. (2009) Disordered macrophage cytokine secretion underlies impaired acute inflammation and bacterial clearance in Crohn's disease. J. Exp. Med. 206, 1883–1897 10.1084/jem.20091233 19652016 PMC2737162

[160] Kane, M., Zang, T.M., Rihn, S.J., Zhang, F., Kueck, T., Alim, M. et al. (2016) Identification of interferon-stimulated genes with antiretroviral activity. Cell Host Microbe 20, 392–405 10.1016/j.chom.2016.08.005 27631702 PMC5026698

[161] Murakami, Y., Hoshi, M., Imamura, Y., Arioka, Y., Yamamoto, Y. and Saito, K. (2013) Remarkable role of indoleamine 2,3-dioxygenase and tryptophan metabolites in infectious diseases: potential role in macrophage-mediated inflammatory diseases. Mediat. Inflamm. 2013, 391984 10.1155/2013/391984 PMC3588179 23476103

[162] Munn, D.H., Shafizadeh, E., Attwood, J.T., Bondarev, I., Pashine, A. and Mellor, A.L. (1999) Inhibition of T cell proliferation by macrophage tryptophan catabolism. J. Exp. Med. 189, 1363–1372 10.1084/jem.189.9.1363 10224276 PMC2193062

[163] Wang, Z., Gao, Y., Zhang, C., Hu, H., Guo, D., Xu, Y. et al. (2017) Quinolinate phosphoribosyltransferase is an antiviral host factor against hepatitis C virus infection. Sci. Rep. 7, 5876 10.1038/s41598-017-06254-4 28724915 PMC5517448

[164] Ahel, D., Horejsí, Z., Wiechens, N., Polo, S.E., Garcia-Wilson, E., Ahel, I. et al. (2009) Poly(ADP-ribose)-dependent regulation of DNA repair by the chromatin remodeling enzyme ALC1. Science 325, 1240–1243 10.1126/science.1177321 19661379 PMC3443743

[165] Gottschalk, A.J., Timinszky, G., Kong, S.E., Jin, J., Cai, Y., Swanson, S.K. et al. (2009) Poly(ADP-ribosyl)ation directs recruitment and activation of an ATP-dependent chromatin remodeler. Proc. Natl Acad. Sci. U.S.A. 106, 13770–13774 10.1073/pnas.0906920106 19666485 PMC2722505

[166] Timinszky, G., Till, S., Hassa, P.O., Hothorn, M., Kustatscher, G., Nijmeijer, B. et al. (2009) A macrodomain-containing histone rearranges chromatin upon sensing PARP1 activation. Nat. Struct. Mol. Biol. 16, 923–929 10.1038/nsmb.1664 19680243

[167] Chen, D., Vollmar, M., Rossi, M.N., Phillips, C., Kraehenbuehl, R., Slade, D. et al. (2011) Identification of macrodomain proteins as novel O-acetyl-ADP-ribose deacetylases. J. Biol. Chem. 286, 13261–13271 10.1074/jbc.M110.206771 21257746 PMC3075673

[168] Jankevicius, G., Hassler, M., Golia, B., Rybin, V., Zacharias, M., Timinszky, G. et al. (2013) A family of macrodomain proteins reverses cellular mono-ADP-ribosylation. Nat. Struct. Mol. Biol. 20, 508–514 10.1038/nsmb.2523 23474712 PMC7097781

[169] Rosenthal, F., Feijs, K.L.H., Frugier, E., Bonalli, M., Forst, A.H., Imhof, R. et al. (2013) Macrodomain-containing proteins are new mono-ADP-ribosylhydrolases. Nat. Struct. Mol. Biol. 20, 502–507 10.1038/nsmb.2521 23474714

[170] Slade, D., Dunstan, M.S., Barkauskaite, E., Weston, R., Lafite, P., Dixon, N. et al. (2011) The structure and catalytic mechanism of a poly(ADP-ribose) glycohydrolase. Nature 477, 616–620 10.1038/nature10404 21892188 PMC3184140

[171] Abraham, R., Hauer, D., McPherson, R.L., Utt, A., Kirby, I.T., Cohen, M.S. et al. (2018) ADP-ribosyl-binding and hydrolase activities of the alphavirus nsP3 macrodomain are critical for initiation of virus replication. Proc. Natl Acad. Sci. U.S.A. 115, e10457–e10466 10.1073/pnas.1812130115 30322911 PMC6217424

[172] Abraham, R., McPherson, R.L., Dasovich, M., Badiee, M., Leung, A.K.L. and Griffin, D.E. (2020) Both ADP-ribosyl-binding and hydrolase activities of the alphavirus nsP3 macrodomain affect neurovirulence in mice. MBio 11, e03253-19 10.1128/mBio.03253-19 32047134 PMC7018654

[173] Alhammad, Y.M.O. and Fehr, A.R. (2020) The viral macrodomain counters host antiviral ADP-ribosylation. Viruses 12, 384 10.3390/v12040384 PMC7232374 32244383

[174] Fehr, A.R., Athmer, J., Channappanavar, R., Phillips, J.M., Meyerholz, D.K. and Perlman, S. (2015) The nsp3 macrodomain promotes virulence in mice with coronavirus-induced encephalitis. J. Virol. 89, 1523–1536 10.1128/jvi.02596-14 25428866 PMC4300739

[175] McPherson, R.L., Abraham, R., Sreekumar, E., Ong, S.E., Cheng, S.J., Baxter, V.K. et al. (2017) ADP-ribosylhydrolase activity of Chikungunya virus macrodomain is critical for virus replication and virulence. Proc. Natl Acad. Sci. U.S.A. 114, 1666–1671 10.1073/pnas.1621485114 28143925 PMC5321000

[176] Parvez, M.K. (2015) The hepatitis E virus ORF1 ‘X-domain’ residues form a putative macrodomain protein/Appr-1″-pase catalytic-site, critical for viral RNA replication. Gene 566, 47–53 10.1016/j.gene.2015.04.026 25870943 PMC7127128

[177] Rajman, L., Chwalek, K. and Sinclair, D.A. (2018) Therapeutic potential of NAD-boosting molecules: the in vivo evidence. Cell Metab. 27, 529–547 10.1016/j.cmet.2018.02.011 29514064 PMC6342515

[178] Yoshino, J., Baur, J.A. and Imai, S.I. (2018) NAD(+) intermediates: the biology and therapeutic potential of NMN and NR. Cell Metab. 27, 513–528 10.1016/j.cmet.2017.11.002 29249689 PMC5842119

[179] Mills, K.F., Yoshida, S., Stein, L.R., Grozio, A., Kubota, S., Sasaki, Y. et al. (2016) Long-term administration of nicotinamide mononucleotide mitigates age-associated physiological decline in mice. Cell Metab. 24, 795–806 10.1016/J.CMET.2016.09.013 28068222 PMC5668137

[180] Cantó, C., Houtkooper, R.H., Pirinen, E., Youn, D.Y., Oosterveer, M.H., Cen, Y. et al. (2012) The NAD(+) precursor nicotinamide riboside enhances oxidative metabolism and protects against high-fat diet-induced obesity. Cell Metab. 15, 838–847 10.1016/J.CMET.2012.04.022 22682224 PMC3616313

[181] Martens, C.R., Denman, B.A., Mazzo, M.R., Armstrong, M.L., Reisdorph, N., McQueen, M.B. et al. (2018) Chronic nicotinamide riboside supplementation is well-tolerated and elevates NAD^+^ in healthy middle-aged and older adults. Nat. Commun. 9, 1286 10.1038/S41467-018-03421-7 29599478 PMC5876407

[182] Remie, C.M.E., Roumans, K.H.M., Moonen, M.B.P., Connell, N.J., Havekes, B., Mevenkamp, J. et al. (2020) Nicotinamide riboside supplementation alters body composition and skeletal muscle acetylcarnitine concentrations in healthy obese humans. Am. J. Clin. Nutr. 112, 413–426 10.1093/AJCN/NQAA072 32320006 PMC7398770

[183] Dollerup, O.L., Chubanava, S., Agerholm, M., Søndergård, S.D., Altıntaş, A., Møller, A.B. et al. (2020) Nicotinamide riboside does not alter mitochondrial respiration, content or morphology in skeletal muscle from obese and insulin-resistant men. J. Physiol. 598, 731–754 10.1113/JP278752 31710095

[184] Yoshino, M., Yoshino, J., Kayser, B.D., Patti, G.J., Franczyk, M.P., Mills, K.F. et al. (2021) Nicotinamide mononucleotide increases muscle insulin sensitivity in prediabetic women. Science 372, 1224–1229 10.1126/SCIENCE.ABE9985 33888596 PMC8550608

[185] Irie, J., Inagaki, E., Fujita, M., Nakaya, H., Mitsuishi, M., Yamaguchi, S. et al. (2020) Effect of oral administration of nicotinamide mononucleotide on clinical parameters and nicotinamide metabolite levels in healthy Japanese men. Endocr. J. 67, 153–160 10.1507/endocrj.EJ19-0313 31685720

