# Correction: NAD^+^-consuming enzymes in immune defense against viral infection

**DOI:** 10.1042/BCJ-479-581

**Published:** 2022-03-04

The authors of the original article “NAD^+^-consuming enzymes in immune defense against viral infection” (*Biochem J.* (2021) **478**:4071-4092) would like to correct an error in Table 1. The authors state that the first column of Table 1 was accidentally deleted during the revision process of their manuscript. The corrected Table 1 is included in this Correction. The authors apologise for this oversight and any inconvenience it may have caused.

**Table 1.** Summary of PARP role in viral infections

PARP	Positive role for host	Ref	Negative role for host	Ref
	Adeno associated virus (AAV)	36	HIV-1	41–45
	Kaposi sarcoma-associated herpesvirus (KSHV)	34	Influenza A virus (IAV)	50, 51
	Epstein-Barr virus (EBV)	37		
PARP1	Hepatitis B virus (HBV)	38		
	Human immunodeficiency virus 1 (HIV-1)	46, 52		
	Murine leukemia virus (MLV)	46		
	Murine gammaherpesvirus 68 (MHV-68)	39, 40		
PARP5a/b	EBV	55	Herpes simplex virus (HSV-1)	53
	Sindbis virus (SINV)	61	Murine coronavirus (MHV)	62
PARP7	Rubella virus	61		
	Venezuelan equine encephalitis virus (VEEV)	60		
	Encephalomyocarditis virus (EMCV)	70		
	IAV	71		
PARP9	SINV	71	N/A	
	Retrovirus	71		
	VSV	71		
PARP10	Avian influenza virus (AIV)	72	N/A	
PARP11	Zika virus	74	VSV	73
			HSV-1	73
	Vesicular stomatitis virus (VSV)			
	MHV-68	78		
	VEEV	80, 82		
	SINV	80		
PARP12	Encephalomyocarditis virus (EMCV)	80	N/A	
	Rift valley fever virus (RVFV)	80		
	Chikungunya virus (CHIKV)	80		
	Zika virus	81		
	MHV	62		
	Human T cell leukemia type 1 (HTLV-1)	85		
	Japanese encephalitis (JE) virus	86		
	Porcine reproductive and respiratory syndrome virus (PRRS)	87		
	IAV	88, 89		
PARP13	HBV	89	N/A	
	HIV-1	90		
	MHV-68	91		
	Alphavirus	92		
	Newcastle disease virus (NDV)	98		
	SARS-CoV-2	103		
PARP14	Human CoV-229E	62		
	MHV	62	N/A	

